# An Extracellular Siderophore Is Required to Maintain the Mutualistic Interaction of *Epichloë festucae* with *Lolium perenne*

**DOI:** 10.1371/journal.ppat.1003332

**Published:** 2013-05-02

**Authors:** Linda J. Johnson, Albert Koulman, Michael Christensen, Geoffrey A. Lane, Karl Fraser, Natasha Forester, Richard D. Johnson, Gregory T. Bryan, Susanne Rasmussen

**Affiliations:** 1 AgResearch Limited, Grasslands Research Centre, Palmerston North, New Zealand; 2 Lipid Profiling and Signalling Group, MRC Human Nutrition Research, Elsie Widdowson Laboratory, Cambridge, United Kingdom; University of Melbourne, Australia

## Abstract

We have identified from the mutualistic grass endophyte *Epichloë festucae* a non-ribosomal peptide synthetase gene (*sidN*) encoding a siderophore synthetase. The enzymatic product of SidN is shown to be a novel extracellular siderophore designated as epichloënin A, related to ferrirubin from the ferrichrome family. Targeted gene disruption of *sidN* eliminated biosynthesis of epichloënin A *in vitro* and *in planta*. During iron-depleted axenic growth, Δ*sidN* mutants accumulated the pathway intermediate N^5^-*trans*-anhydromevalonyl-N^5^-hydroxyornithine (*trans*-AMHO), displayed sensitivity to oxidative stress and showed deficiencies in both polarized hyphal growth and sporulation. Infection of *Lolium perenne* (perennial ryegrass) with Δ*sidN* mutants resulted in perturbations of the endophyte-grass symbioses. Deviations from the characteristic tightly regulated synchronous growth of the fungus with its plant partner were observed and infected plants were stunted. Analysis of these plants by light and transmission electron microscopy revealed abnormalities in the distribution and localization of Δ*sidN* mutant hyphae as well as deformities in hyphal ultrastructure. We hypothesize that lack of epichloënin A alters iron homeostasis of the symbiotum, changing it from mutually beneficial to antagonistic. Iron itself or epichloënin A may serve as an important molecular/cellular signal for controlling fungal growth and hence the symbiotic interaction.

## Author Summary

Perennial ryegrass is a cool-season grass that is agriculturally important as forage, especially in Australasia. Essential to the longevity and maintenance of healthy pastures are epichloae fungi, such as *Epichloë/Neotyphodium* spp. that live inside these grasses in a symbiotic manner. These fungi produce bioactive compounds that can protect its host grass from various abiotic and biotic stresses. They depend on resource supply by their host, including nutrients that are taken up by the roots of the grass host. We have found that iron, an indispensable element for growth, is acquired by *Epichloë festucae* from its grass host via the secretion of the extracellular siderophore epichloënin A. Without iron-mediated siderophore uptake to capture host iron, the symbiotic relationship becomes disturbed and the growth habit of the fungus is no longer restricted. Our results indicate that secretion of epichloënin A enables the symbiotic fungus to compete for plant iron while avoiding fungal over-growth inside the host plant.

## Introduction

Iron is an essential nutrient for almost all organisms (except for some lactobacilli [1]) due to its central role in vital cellular reactions. The redox properties of iron confer a catalytic function essential for fundamental metabolic pathways such as DNA synthesis, respiration and photosynthesis [2], [3]. Even though iron is a highly abundant metal, in aerobic environments bioavailability is low because ferric iron forms insoluble oxides through reaction with oxygen. Iron concentrations in tissues must also be carefully regulated since free iron is deleterious given that it has the potential to catalyze the production of cytotoxic reactive oxygen species (ROS) through the Haber-Weiss/Fenton reactions [4]. Controlling iron homeostasis is therefore an essential function and organisms have developed complex strategies for iron uptake, utilization, transport and storage [5]. The uptake of iron is presumably the principal regulatory point of iron homeostasis and multiple high- and low-affinity iron uptake systems have evolved [6].

In mammals and plants excess iron is tightly sequestered by high-affinity binding proteins [7], [8], and deliberately withdrawing iron is a documented defense strategy employed by animal hosts against invading bacterial pathogens in order to limit their growth [9], [10]. For microbes to acquire limited iron from animal or plant hosts, high affinity iron uptake systems are required. One such mechanism is siderophore-mediated iron uptake. Under iron starvation, most fungi and bacteria synthesize siderophores, low molecular weight (M_r_<1,500) iron-chelating agents, to solubilize ferric iron and control their intracellular iron levels [11]–[13]. The functional role of siderophores may be extracellular – secreted as iron-free siderophores to chelate iron for cellular iron uptake; and/or intracellular – located within the cell for intracellular iron storage. A number of roles have been attributed to fungal siderophores and these include functions such as virulence factors and asexual/sexual determinants [14]–[21]. Another major type of high affinity iron acquisition in fungi is reductive iron assimilation (RIA) which is based on a ferroxidation/permeation uptake [6], [22], [23]. Some fungi are capable of utilizing both of these high affinity uptake systems; examples are *Ustilago maydis*, *Aspergillus fumigatus* and *Fusarium graminearum* [15], [16], [24]–[28]. Nevertheless, characterization studies of both siderophore and RIA uptake systems in the same fungal species through respective gene deletion studies have shown that only one of these two systems is essential for mammalian or plant disease by a given fungal pathogen and which one is indispensable thus appears to be fungal species dependent and possibly related to their lifestyles. Examples of fungi demonstrated to require the siderophore system for virulence are *A. fumigatus*, *Histoplasma capsulatum* and *F. graminearum* [16], [19], [29], whereas RIA is essential for the virulence of the basidomycetes *U. maydis*, *Candida albicans* and *Cryptococcus neoformans* [15], [30], [31].

Nearly all fungi produce hydroxamate-type siderophores [32], which are typically composed of three hydroxamate groups linked by peptide or ester bonds to form an octahedral complex. Siderophores are classified into four major structural types: ferrichromes, fusarinines, coprogens and rhodotorulic acid [12]. Their formation by the linkage of hydroxamate groups or additional amino acids in the case of ferrichrome-type siderophores is catalyzed by non-ribosomal peptide synthetases (NRPS) [28]. NRPSs are multifunctional enzymes that synthesize peptides by a thio-template mechanism and are modular in structure. A typical module consists of an adenylation (A) domain for substrate specificity, a peptidyl carrier domain (T), which binds a 4′-phosphopantetheine cofactor, and a condensation (C) domain for bond formation [33]. NRPS genes functionally identified to encode fungal siderophore synthetases with an extracellular role are *sid2* and *fer3* of *U. maydis* and *sib1* of *Schizosacccharomyces pombe*, involved in the synthesis of ferrichrome-type siderophores [28], [34], [35], *sidD* from *A. fumigatus* involved in triacetylfusarinine C (TAFC) production [36] as well as *NPS6* of *Alternaria brassicicola*, *F. graminearum*, and *Cochliobolus heterostrophus* involved in the respective production of N^α^-dimethylcoprogen, TAFC and coprogens [19]. *NPS6* deletion studies have demonstrated that these extracellular siderophores play a role in fungal virulence to plants [19]. Recently, another *NPS6* ortholog responsible for the production of coprogens was identified from the plant pathogenic fungus *Magnaporthe grisea*, but loss of the corresponding gene did not affect virulence in rice [37], whereas the gene encoding the NRPS responsible for the synthesis of the intracellular siderophore, ferricrocin, was required for full virulence of *M. grisea* [20]. Fungi that form mutualistic relationships with plants such as the widespread symbioses of mycorrhizal fungi with terrestrial plant communities also produce siderophores [38]–[40]. In contrast to fungal pathogens, siderophore production by mycorrhizal fungi has been postulated to aid their hosts by improving solubilization of insoluble iron oxides resulting in a positive effect on the iron nutrition of the host plant [41].

The epichloae fungi of family Clavicipitaceae (comprising genera *Epichloë* and *Neotyphodium*) form symbioses with temperate grasses of the subfamily Poöideae [42]. These Poöideae-epichloae associations comprise an evolutionary continuum from mutualistic to antagonistic, with the nature of this relationship determined by the importance of vertical (via host seeds) versus horizontal (ascospore mediated) transmission of the fungus [42]. During colonisation of grass leaves, the endosymbiont's growth is tightly regulated and synchronized with the growth of its host plant [43]. Fungal hyphae are confined to the intercellular spaces of leaf sheaths and blades where the endophyte induces no symptoms [44]. Epichloid symbioses can be mutually beneficial with the plant providing the endophyte with nutrients, and the endophyte conferring biotic and abiotic advantages to the host plant [44]–[48]. Improved herbivore resistance of infected plants is linked to the production of fungal secondary metabolites (alkaloids), and it appears that the host plant plays a key role in the regulation of some of these metabolites [49], [50]. In a study to investigate the distribution and diversity of NRPS gene families within the epichloae, two NRPS fragments, *NRPS2* and *NRPS9*, were isolated with proposed functions identified as siderophore-like [51]. Our unpublished data shows that *NRPS9* encodes *sidC*, the siderophore synthetase for ferricrocin (L. Johnson et al., unpublished results). *NRPS2*, renamed as *sidN*, was confirmed to be a siderophore-synthesizing NRPS from three-dimensional structural elucidation and biochemical studies of the third adenylation domain of SidN [52]. These studies showed that this domain activates anhydromevalonyl-N^6^-hydroxy-_L_-ornithine (AMHO), a large hydroxamate amino acid known to be a component of fungal siderophores [52]. More recently, the structure of a novel ferrichrome-type siderophore containing AMHO moieties which we have designated as epichloenin A, has been elucidated by high resolution tandem mass spectrometry (HRMSMS) and NMR from iron-depleted cultures of *E. festucae* [53]. An additional minor variant, epichloënin B, has also been detected in cultures of WT and C-*sidN* strains [53]. Here, we report a study establishing that *sidN* (formerly *NRPS2*) encodes a siderophore synthetase required for the biosynthesis of the extracellular siderophore, epichloënin A. Our research shows that *sidN* is required for maintaining the mutualistic interaction of the endophyte *E. festucae* with perennial ryegrass.

## Results

### *sidN* Encodes a Siderophore Synthetase Involved in the Biosynthesis of the Extracellular Siderophore, Epichloënin A

To investigate whether *sidN* (formally *NRPS2*) is involved in the biosynthesis of epichloënin A and its role in endophyte-grass symbioses, a gene disruption construct was designed and the gene disruption performed by homologous recombination in *E. festucae* wild-type (WT) strain Fl1 (see Methods for details). Four independent *ΔsidN* mutants (*ΔsidN* 85, 82, 54, and 26) were identified at a homologous recombination frequency of 10%. These four mutants displayed similar phenotypes as reported throughout this study.

**Figure 1:**
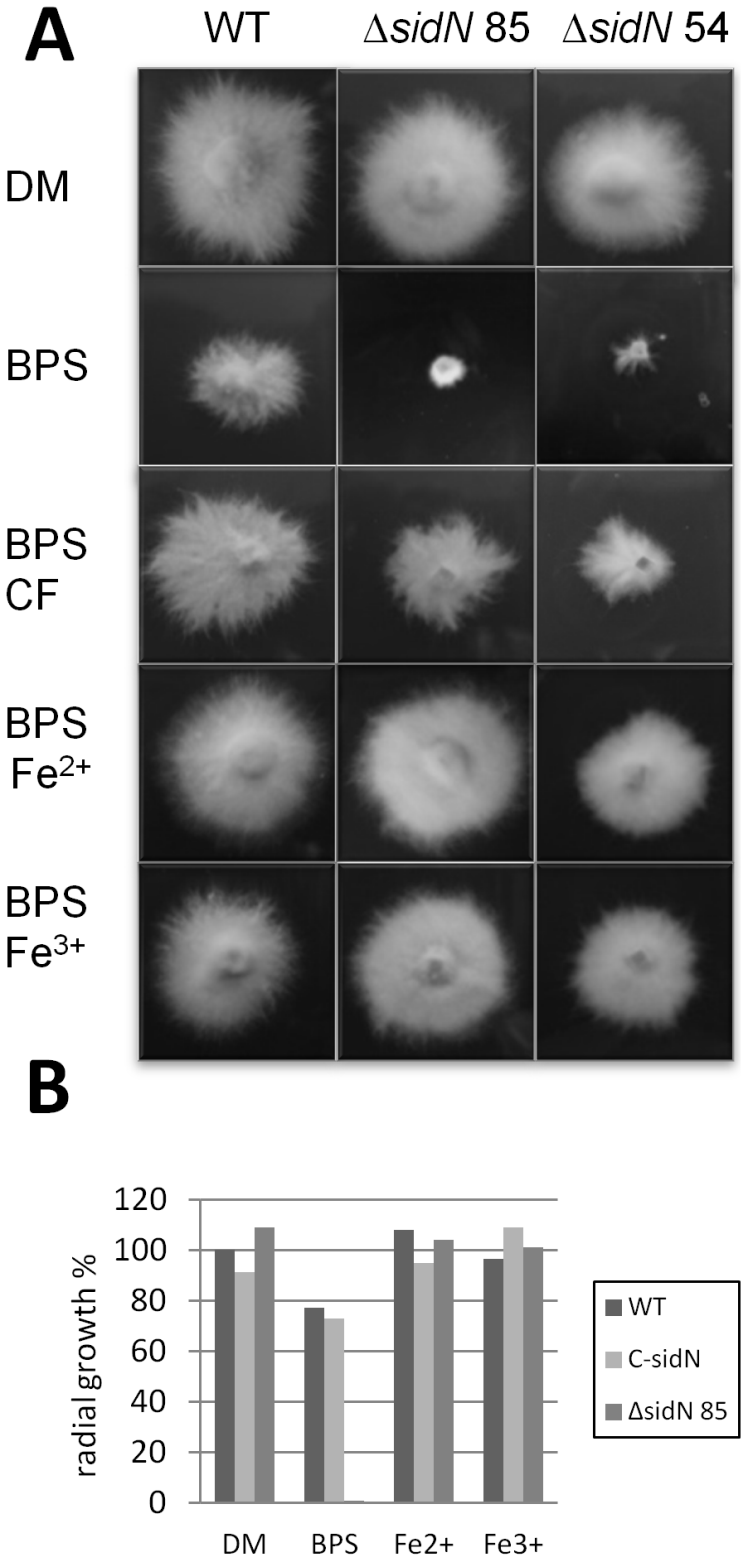
Iron depletion renders Δ*sidN* mutants incapable of axenic vegetative growth. A. 16-day-old cultures of wild-type *E. festucae* Fl1 (WT) and Δ*sidN* mutant strains (*ΔsidN* 54 and *ΔsidN* 85) were grown on iron depleted defined media (DM), and DM media supplemented with 100 µM BPS, or 100 µM BPS and culture filtrate from WT (BPS CF), or 100 µM BPS and 20 µM FeS0_4_ (BPS Fe^2+^) or 100 µM BPS and 20 µM FeCl_3_ (BPS Fe^3+^) respectively. B. Radial growth measurements of WT, complement (C-sidN) and Δ*sidN* 85 were determined by inoculating mycelial plugs in triplicate onto DM media, or DM supplemented with 100 µM BPS, DM with 100 µM BPS and 20 µM FeS0_4_ (Fe2+) and DM with 100 µM BPS and 20 µM FeCl_3_ (Fe3+) respectively. Colonies were measured at 10 days. The results represent the mean of three independent experiments. The radial growth is normalized to that of WT grown on DM media.

The axenic vegetative growth characteristics of the Δ*sidN E. festucae* mutants were examined on defined medium (DM) and on DM supplemented with the ferrous iron chelator bathophenanthrolinedisulfonic acid (BPS), which is impermeable to cell membranes [54], [55] (Figure 1A). BPS supplementation sequesters trace ferrous iron from the extracellular medium, subsequently inhibiting RIA iron uptake by removing the substrate for this process. For the WT, addition of BPS to DM moderately reduced the radial growth rate (by approximately 20%), whereas radial growth of the Δ*sidN* mutants was almost completely inhibited (shown for two independent Δ*sidN* mutants in Figure 1A). The lack of growth by the mutants on this medium is explainable by the loss of both RIA-mediated (via ferrous iron BPS chelation) and siderophore mediated iron uptake from the extracellular medium (i.e. loss of biosynthesis of the extracellular siderophore), and demonstrates the presence of RIA in *E. festucae*. The inhibition of radial growth on DM+BPS medium could be moderately complemented by the addition of enriched culture supernatants (Sephadex-column purified culture filtrate - CF) from WT (Figure 1A), but not from Δ*sidN* 85 (data not shown), indicating the presence of the native extracellular siderophore in WT CF only. Growth of all Δ*sidN* mutant strains could also be fully restored by addition of FeSO_4_, or FeCl_3_ to DM+BPS medium (Figure 1A).

**Figure 2:**
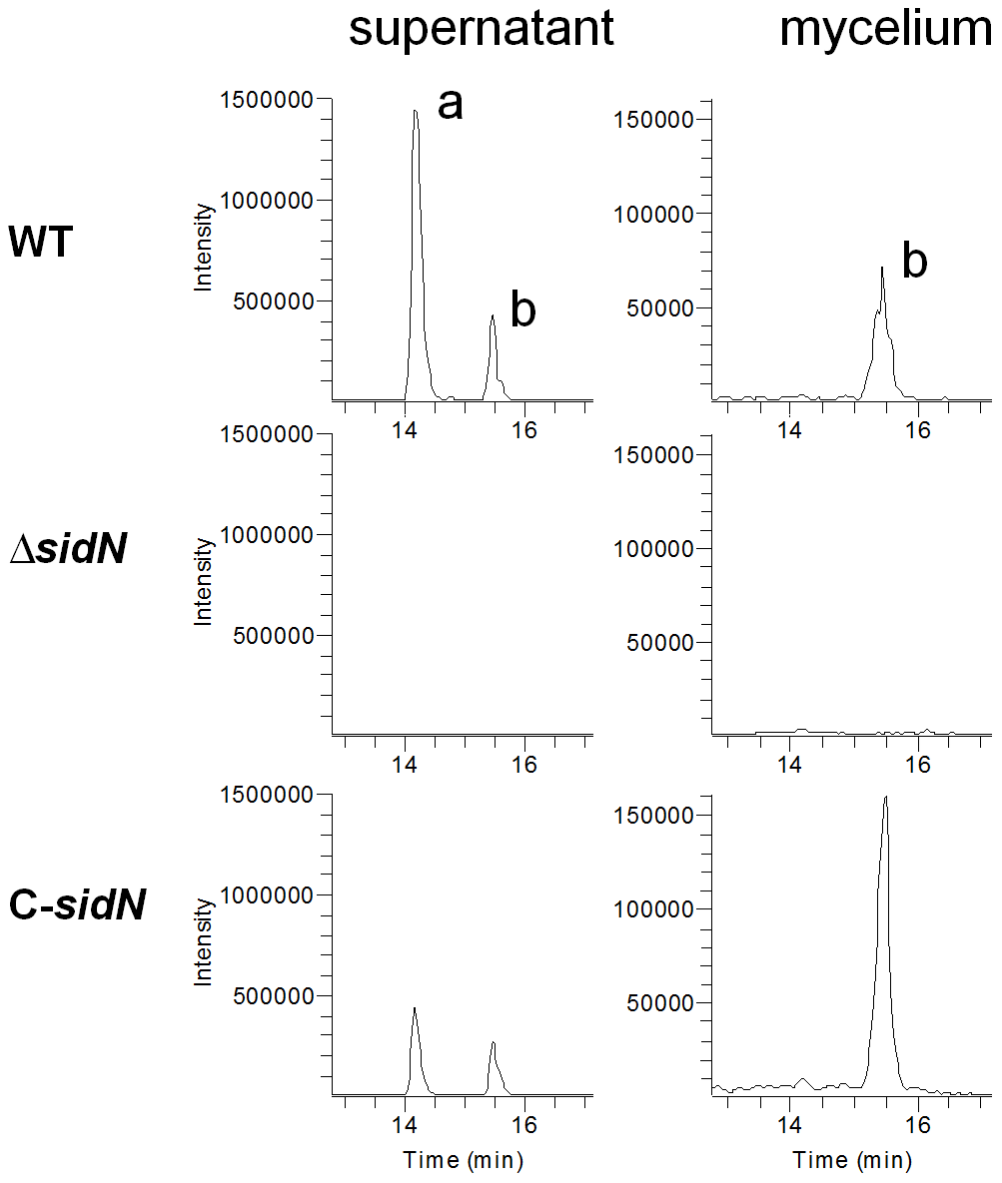
Synthesis of epichloënin A is dependent on *sidN*. LC-MS analysis showing MS^1^extracted ion chromatograms for both epichloënin A (a, *m/z* 542) and ferriepichloënin A (b, *m/z* 569) in supernatant and mycelium from two week old iron-depleted cultures of wild-type *E. festucae* Fl1 (WT), *ΔsidN* mutant 85 (*ΔsidN*), and a complemented *ΔsidN* strain (C-*sidN*). Note scale for supernatant is 10× of that for mycelium.

**Figure 3:**
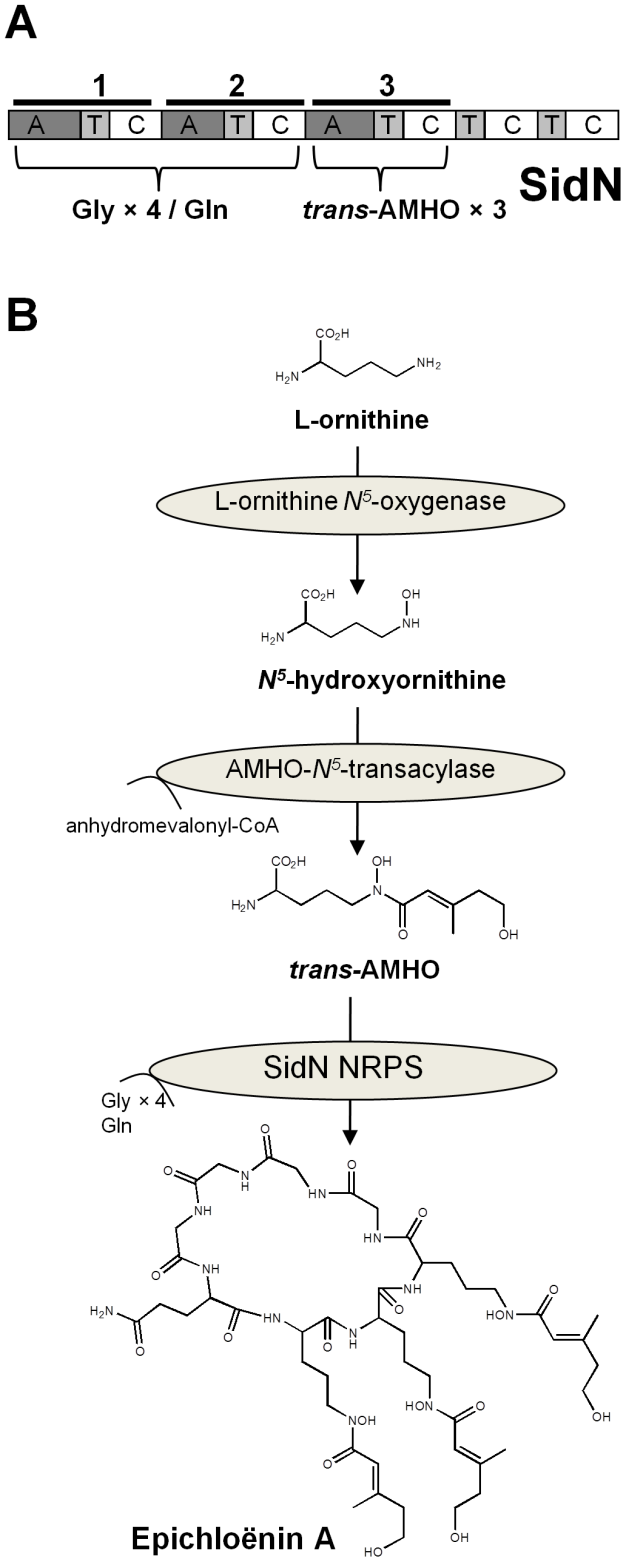
SidN modular structure and postulated siderophore biosynthetic pathway for epichloënin A. A. NRPS enzyme, SidN assembles Epichloënin A by activating and incorporating three *trans*-anhydromevalonylhydroxyornithine (*tran*s-AMHO), 1 glutamine and 4 glycine moieties. This pathway is according to Plattner and Diekmann [56]. B. The three modules of SidN are composed of an A (adenylation) domain, a T (peptidyl carrier) domain and a C (condensation) domain. Proposed substrates for modules 1 and 2 are glycine (Gly) and glutamine (Gln) residues, while for module 3 are *trans-*AMHO residues.

To validate that the phenotypic effects described in the *ΔsidN* strains arose from gene inactivation of *sidN*, a complementation was performed by co-transforming protoplasts from *ΔsidN* 85 with fosmid clone G8-5 (containing the full-length genomic copy of *E. festucae sidN*) and a plasmid containing the geneticin resistance gene (pII99). Screening for complementation was carried out using an *in vitro* plate assay that exploited the phenotypic trait of the Δ*sidN* mutants to have extremely poor mycelial growth on BPS-containing, iron-depleted DM (see Figure 1A). Four independent transformants formed large colonies on BPS medium. We designated the complemented strain used for further studies C-*sidN*; measurements of radial growth of Δ*sidN* 85, WT and C-*sidN* show that C-*sidN* is able to grow at 94% of WT on BPS supplemented DM (Figure 1B). This indicates that transformation with the full-length genomic copy of *sidN* restored growth of Δ*sidN* mutants on BPS supplemented medium and congruent with *sidN* encoding a biosynthetic enzyme for high-affinity siderophore uptake of iron from extremely iron-depleted medium. To determine if the siderophore assembled by the biosynthetic pathway involving SidN was epichloënin A, a chemical analysis was performed on cultures of the WT, *ΔsidN* mutant and complemented C-*sidN* strains grown under iron depleted conditions (Figure 2). Culture supernatant and mycelial extracts from these strains were analyzed by liquid chromatography – mass spectrometry (LCMS). Epichloënin A (MS^1^ *m/z* 542 ([MH_2_]^2+^) was the predominant form in the supernatant of WT cultures (Figure 2), while ferriepichloënin A (MS^1^ *m/z* 569 ([MH_2_]^2+^) predominated in the mycelial extracts. Since the ratio of the iron-free to iron-bound form is much higher in the extracellular supernatant than in the mycelium, we conclude that epichloënin A is an extracellular siderophore. A similar pattern of occurrence was observed for the C-*sidN* supernatants, albeit at reduced concentrations compared to WT. However, both forms were absent from supernatants and mycelia of the *ΔsidN* mutant strains, clearly demonstrating that SidN is responsible for the assembly of epichloënin A. Figure 3A presents the postulated siderophore biosynthetic pathway according to Plattner and Diekmann [56] adapted for epichloënin A biosynthesis in *E. festucae*.

### The Modular Structure of SidN and Genome Analyses

To enable further investigation of *sidN* (formally *NRPS2*), including the determination of the modular architecture of this siderophore-synthesizing NRPS, we obtained the full-length gene sequence by constructing an *E. festucae* (strain Fl1) fosmid library. Screening of this library by PCR (using *NRPS2* specific primers) gave three positive clones, of which one (clone G8-5) contained the entire genomic copy of the full-length gene. Sequencing of the *E. festucae* Fl1 *sidN* fosmid clone revealed an open reading frame (ORF) of 14,073 bp with no introns. A BLASTX analysis of the deduced amino acid sequence of *sidN* revealed similarities to fungal NRPS genes involved in siderophore biosynthesis. Top hits to SidN (with an expect value of 0.0) included functionally characterized siderophore NRPSs involved in the biosynthesis of ferricrocin or ferrichrome. The top hit was to *A. fumigatus* SidC [36] at 34% amino acid identity (52% positives). Out of the six modular architectures described for ferrichrome synthetase NRPSs by Bushley et al. [57], the most significant hits using *sidN* as the query sequence were to 12 of the 13 Type II ferrichrome NRPSs, followed by less similarity to other ferrichromes. These results support our previous findings that SidN encodes a siderophore biosynthetic enzyme of the ferrichrome family [52], and upholds our current discovery that SidN assembles epichloënin A, a novel ferrichrome family member. It is however interesting that the amino acid sequence analyses indicates SidN has the highest similarity to SidC which assembles ferricrocin, but the acyl groups of the hydroxamates of both epichloënin A and ferricrocin are different: for SidN it is *trans*- anhydromevalonyl [52], [53], whereas for ferricrocin, a simple acetyl group is incorporated into its hydroxamate group [11].

Analysis of the SidN amino acid sequence revealed three complete A-T-C modules and a terminal T-C repeat identical to Type II ferrichrome NRPSs (where A = adenylation domain, T = peptidyl carrier domain and C = condensation domain) (Figure 3B). From the work by Lee et al. [52] the third domain (A3) of SidN incorporates the hydroxamate groups of the siderophore which forms an octahedral iron complex. The other component amino acids of epichloënin A are one glutamine and four glycines [53], which we postulate are assembled by SidN A domains one (A1) and two (A2). Analysis of the putative binding pocket residues of the SidN A1 and A2 domains showed that they were dissimilar to those of other characterized fungal siderophore synthetases [28], [57] and did not allow a prediction of which A domain activated glutamine or glycine or the number of iterative cycles of their addition into the peptide. Figure 3B shows that A1 and A2 domains of SidN activate either the addition of one glutamine or four cycles of glycine addition, while the A3 domain is predicted to activate three cycles of addition of *trans*-AMHO residues.

Recently, the genomes of two *E. festucae* strains (Fl1 and E2368) have been sequenced (http://www.endophyte.uky.edu/). A BLASTN analysis of the E2368 and Fl1 *sidN* sequences revealed a 99% nucleotide identity with an expect value of 0.0. We also previously identified a second siderophore-like NRPS from *N. lolii* (*NRPS9*; [51]) which was located on a different supercontig to *sidN* in both *E. festucae* genomes and putatively encodes an intracellular siderophore synthetase, ferricrocin (L. Johnson et al., unpublished results). We have not identified any other siderophore-like NRPS genes in the *E. festucae* Fl1 and E2368 genomes.

To determine what other genes are clustered with *sidN* we analyzed the predicted ORFs surrounding the *sidN* homologue from the *E. festucae* Fl1 and E2368 genome databases. These analyses revealed that just one ORF (*abcI*), located upstream of *sidN* appears to be grouped with *sidN* forming a partial siderophore biosynthetic gene cluster, possibly sharing a divergent promoter with *sidN*. This ORF encodes a putative member belonging to the P-glycoprotein-like multidrug resistance (MDR) subfamily of ATP-binding cassette (ABC) transporters. A similarity search revealed a highly significant hit to ABC1 from *H. capsulatum*, located in the *H. capsulatum* SID1 gene cluster in an analogous position and orientation with respect to its siderophore synthetase, NPS1 [29]. There were no other shared regions of synteny. Of note, *ABC1* is iron-regulated and contains a putative Sre1 GATA motif consensus site, for regulation by the GATA type iron regulator [29].

### *In vitro trans*-AMHO Accumulation

Schrettl et al. [36] reported the accumulation of the siderophore precursor *cis*-AMHO, in the supernatant of an iron-depleted culture of a *sidD* deletion mutant; *sidD* encodes the NRPS responsible for fusarinine C biosynthesis in *A. fumigatus*. We therefore examined supernatants of cultures of *ΔsidN* mutants grown under iron-depleted conditions by LCMSMS for the presence of likely precursors of epichloënin A, and found a product of *m/z* 261 which we identified putatively as *trans*-AMHO by comparison with the retention and MS^2^ spectrum of *cis*-AMHO prepared from fusarinine C [52] (Figure S1A). This is consistent with the finding that epichloënin A incorporates *trans*-AMHO moieties [53]. Targeted LCMSMS analyses of mycelial extracts of the WT, Δ*sidN* 85 mutant and the complemented strain showed that *trans*-AMHO accumulation was very low in the WT, slightly higher in the complemented strain, but nearly 40-fold higher in Δ*sidN* 85 compared to WT, indicating that *trans*-AMHO is a precursor that is significantly accumulating in the mutant (Figure S1B).

### *In vitro* Growth Characteristics of *ΔsidN* Mutants

**Figure 4:**
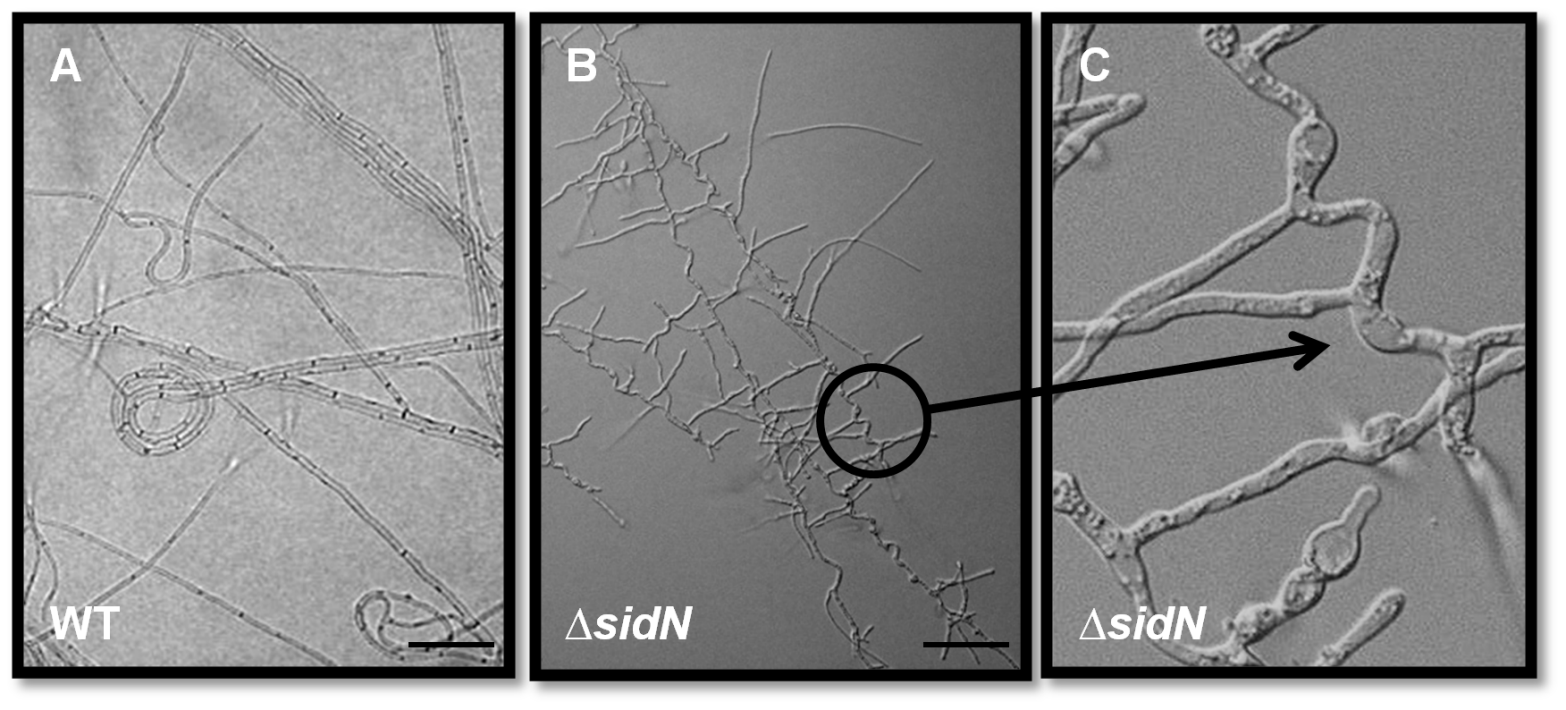
*ΔsidN* mutants display abnormal hyphal morphologies on water-agar. A. Mycelia of wild-type *E. festucae* Fl1 (WT) on water agar. B. Mycelia of *ΔsidN* 85 mutant proliferating on water agar by lateral branches. C. Close up of *ΔsidN* 85 mutant mycelia showing hyphal convolutions and swellings. Bar is 50 µM.

Light microscopy was employed to observe the vegetative hyphal growth characteristics of the *ΔsidN* mutants on water agar as it is a useful medium for studying the morphology of individual hyphal strands within a colony. It is also a nutrient deficient medium. The mutants displayed various abnormalities compared to WT such as increased lateral branching, coils (not shown), atypical hyphal convolutions with a high frequency of hyphal tip and compartmental swellings (Figure 4); complementation in the C-*sidN* strain restored normal hyphal growth (data not shown).

**Figure 5:**
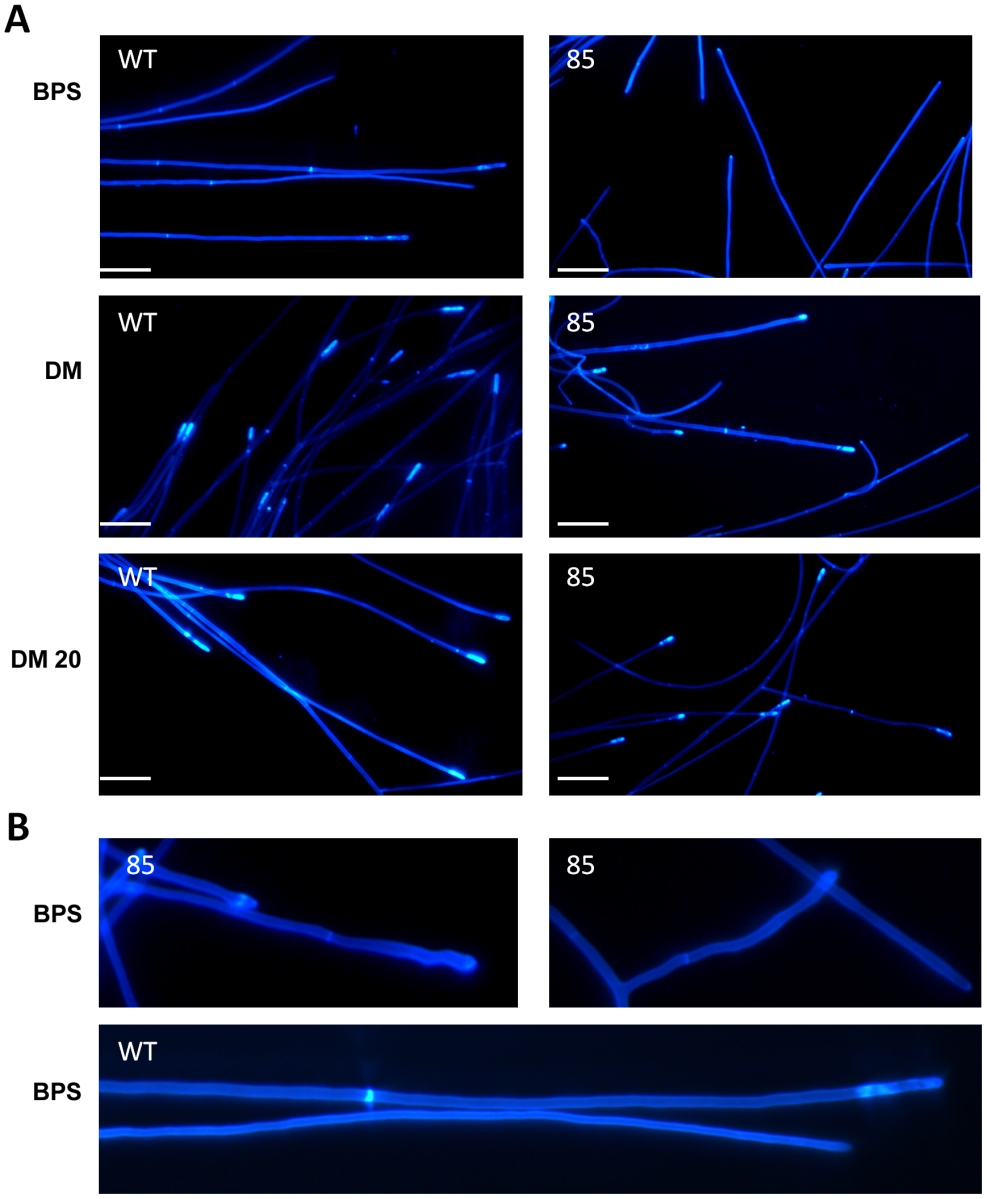
Chitin accumulation is abnormally distributed in Δ*sidN* mutants. A. Chitin distribution pattern in hyphal tips of wild-type *E. festucae* Fl1 (WT) and Δ*sidN* mutant 85 (85) grown in liquid of iron-depleted DM (defined medium) supplemented with BPS (BPS), DM (DM) and DM supplemented with 20 µM Fe^2+^ (DM 20). Bars = 50 µM. B. Enlargement of meandering hyphal tips from A above of wild-type *E. festucae* Fl1 (WT) and Δ*sidN* mutant 85 (85) grown in liquid DM supplemented with BPS (BPS).

To evaluate the cell wall integrity of the *ΔsidN* mutants, staining with Calcofluor White was used to analyze the distribution of chitin (and other β-linked fungal cell wall polysaccharides) [58] in hyphae grown in liquid DM, and DM amended with BPS or 20 µM iron (Figure 5). For the WT, there were no significant differences in tip, septa, or hyphal wall staining in DM or DM supplemented with iron, however, the addition of the ferrous iron chelator BPS to the medium resulted in the majority of tips displaying no or weak speckled staining near the tip region, with septa staining not affected. Also, the hyphal contents appeared to have a much higher background stain compared to staining of hyphae in DM or DM with iron supplementation. The complement was comparable to the WT (results not shown). It is remarkable that addition of BPS, an agent that chelates ferrous iron and as a consequence inhibits RIA uptake, can dramatically alter chitin localization. A similar response to BPS supplementation was observed with stained mycelium from *ΔsidN* 85, yet some specific differences with respect to WT were observed under all 3 conditions tested. In DM, mutant hyphae displayed slightly less tip staining compared to the WT, and often the length of staining at the tip was reduced (Figure 5). Addition of iron appeared to improve overall tip staining length and brightness to that observed for WT hyphae. Supplementation with BPS, also radically affected the chitin distribution of mutant hyphae (similar to WT), however the pattern of hyphal growth on this medium was highly branched (as observed for *ΔsidN* mutants on water agar), and notably some of these branches were extending in a meandering manner (see Figure 5B). Collectively, these observations suggest that the *ΔsidN* mutants are defective in maintaining polar growth when iron availability is low; to our knowledge, this is a feature not previously linked to fungal siderophore mutants.

To test for sensitivity of the *ΔsidN* mutants to oxidative stress, hydrogen peroxide was applied to the growth medium. On complete medium (PD), only *ΔsidN* mutant 85 showed a slight sensitivity to H_2_O_2_, whereas on iron-depleted DM both *ΔsidN* mutants 54 and 85 were significantly more sensitive to H_2_O_2_ than WT (Table 1).

**Table 1:** Colonies of *ΔsidN* mutants are sensitive to hydrogen peroxide on DM.

Growth medium	Fungal strain			Statistics		
	WT	Δ*sidN85*	Δ*sidN54*	LSD	SED	p-value
DM+H_2_O_2_	0.707	0.495	0.52	0.1115	0.0554	0.002
PD+H_2_O_2_	0.878	0.724	0.84	0.1037	0.0408	0.016

Values given are ratios of radial growth measurements of colonies grown for 7 days at 22°C on DM (defined medium) or PD (potato dextrose) medium supplemented with 0.7 mM H_2_O_2_ versus DM or PD. Statistics were generated from an analysis of variance. LSD is the Least Significant Difference between any two means and the SED represents the Standard Error of the Difference.

A reduction in asexual sporulation (in iron-depleted medium) had been previously documented for deletion strains of *C. heterostrophus NPS6* [18]. To study sporulation in the *ΔsidN* mutants, it was necessary to find suitable asexual sporulation conditions to generate sufficient numbers of conidia from the WT strain for comparison. Only growth on water-agar (with a cold-inducing incubation step) produced satisfactory levels of sporulation in the WT on coil structures and hyphal strands, while in comparison, colonies of the *ΔsidN* mutants produced very low numbers of conidia under these conditions (see Figure S2A and S2B). Asexual spore morphology did not appear to be affected by the *sidN* mutation (Figure S2B). It is not currently possible to determine if ascospore formation is affected in these mutants since the pre-sexual choke state required for the commencement of the sexual cycle has never been observed with artificial infection of perennial ryegrass (*L. perenne*) with *E. festucae* [59].

### Iron Represses *sidN* Expression

**Figure 6:**
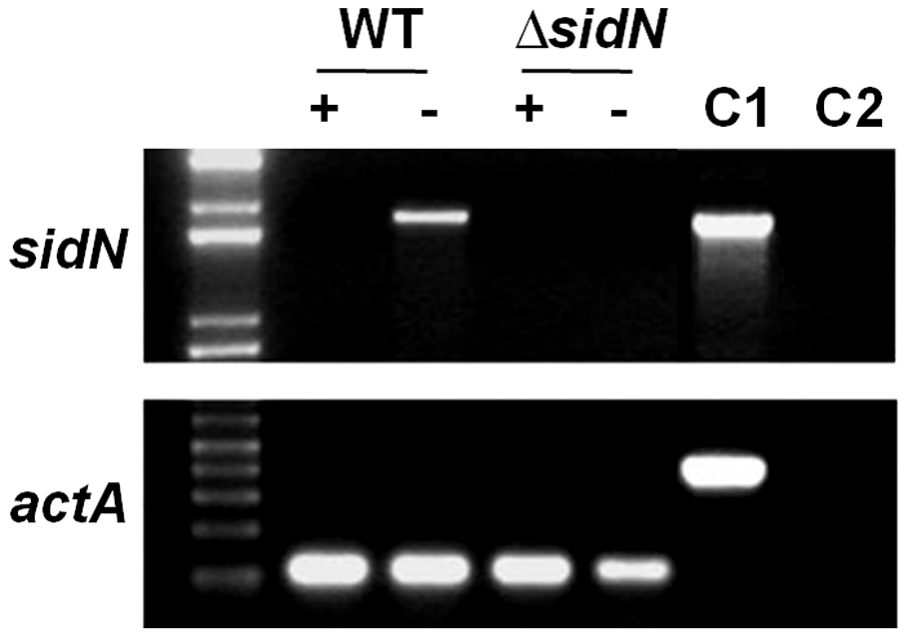
Expression of *sidN* is repressed under iron replete conditions. RT-PCR expression analysis of *ΔsidN* mutant 85 (*ΔsidN*) and wild-type *E. festucae* Fl1 (WT) grown in liquid cultures under iron replete (+) conditions (DM supplemented with 20 µm FeS0_4_) or iron-depleted (−) conditions (DM - no iron supplementation). gDNA is used as a positive control (C1) and water as a negative control (C2) for *sidN* and *actA* (actin) gene expression.

Fungal siderophore biosynthetic genes are typically negatively regulated by iron, and we therefore compared the expression of *sidN* from liquid cultures grown under iron depleted conditions versus iron supplemented conditions. Expression of *sidN* in the WT was strongly repressed under iron supplemented conditions and only detectable by reverse transcriptase polymerase chain reaction (RT-PCR) when grown in iron-depleted medium (Figure 6). As expected, expression of *sidN* was not detected in the *ΔsidN* mutants under any of the conditions tested.

### Loss of *sidN* Adversely Affects the Mutualistic Relationship Between *E. festucae* and Perennial Ryegrass

**Figure 7:**
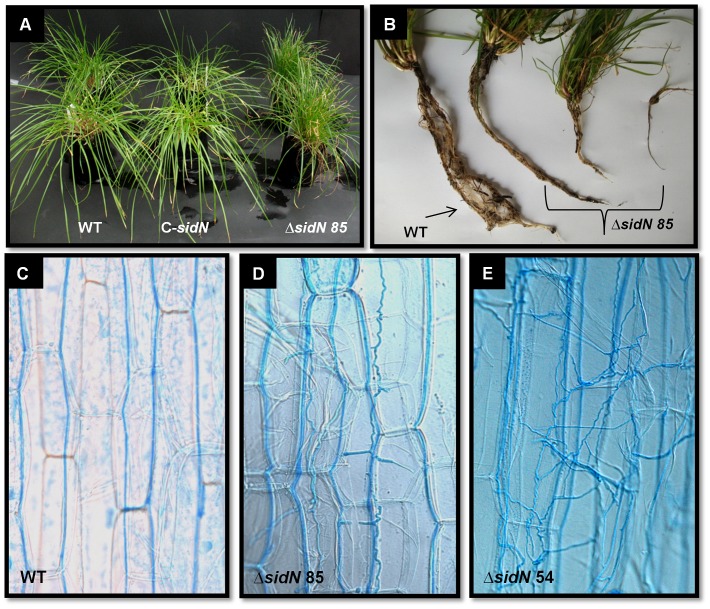
The Endophyte-Grass symbiotic interaction phenotype is disrupted in *ΔsidN* infected plants. A. Phenotypes of perennial ryegrass plants infected with wild-type *E. festucae* Fl1 (WT), complemented *ΔsidN* strain (C-*sidN*) and *ΔsidN* mutant 85 (*ΔsidN*). B. Root systems of *sidN* infected plants are reduced compared to WT infections. A perennial ryegrass plant infected with *E. festucae* (WT) is compared against three Δ*sidN* 85 infected plants displaying increased levels of plant stunting from left to right. Plants are 14 weeks old. C, D and E. Light micrograph DIC images of aniline-blue stained hyphae of wild-type *E. festucae* Fl1 (WT). C. *ΔsidN* mutant 85. D. *ΔsidN* mutant 54. E. in mature leaf sheaths.

To explore the effect of loss of the *sidN* product on the symbiotic relationship of *E. festucae* with perennial ryegrass, *ΔsidN* mutants, WT and C-*sidN* complemented strains were inoculated into perennial ryegrass seedling lines (Figure 7A). Onset of symptom development with the *ΔsidN* mutants was apparent within two weeks of planting inoculated seedlings into soil. Infection with the *ΔsidN* mutants resulted in a range of stunted, erect (versus prostrate) plant phenotypes, all with a large increase in tiller numbers (Figure 7A).

All plants infected with *ΔsidN* mutants showed abnormal underdeveloped root systems, and with the more severely stunted plants having markedly reduced root systems (Figure 7B). Severely stunted *ΔsidN* infected plants frequently died when transplanted from root trainers to larger pots, in contrast to the more moderately stunted plants, which could be maintained for long periods in the glasshouse.

To examine hyphal growth *in planta*, light microscopy of aniline-blue stained leaf sheaths of grass pseudostems was performed (Figure 7C, D, E). Hyphae of WT infected plants grow mainly by intercalary growth typified by infrequently branched hyphae that are generally aligned parallel to the longitudinal leaf axis of the elongating grass leaf [43]. Microscopic examination of *ΔsidN* mutants in perennial ryegrass revealed extensive colonization of leaf sheath tissues in comparison to WT (Figure 7C, D, E). There were large regional areas of synchronized hyphal growth (as described by Christensen et al. [43]) alongside unrestricted convoluted hyphae (Figure 7D, E). Less frequent patches of excessively convoluted hyphae extending out in all directions were also observed (see Figure 7E for an example).

**Figure 8:**
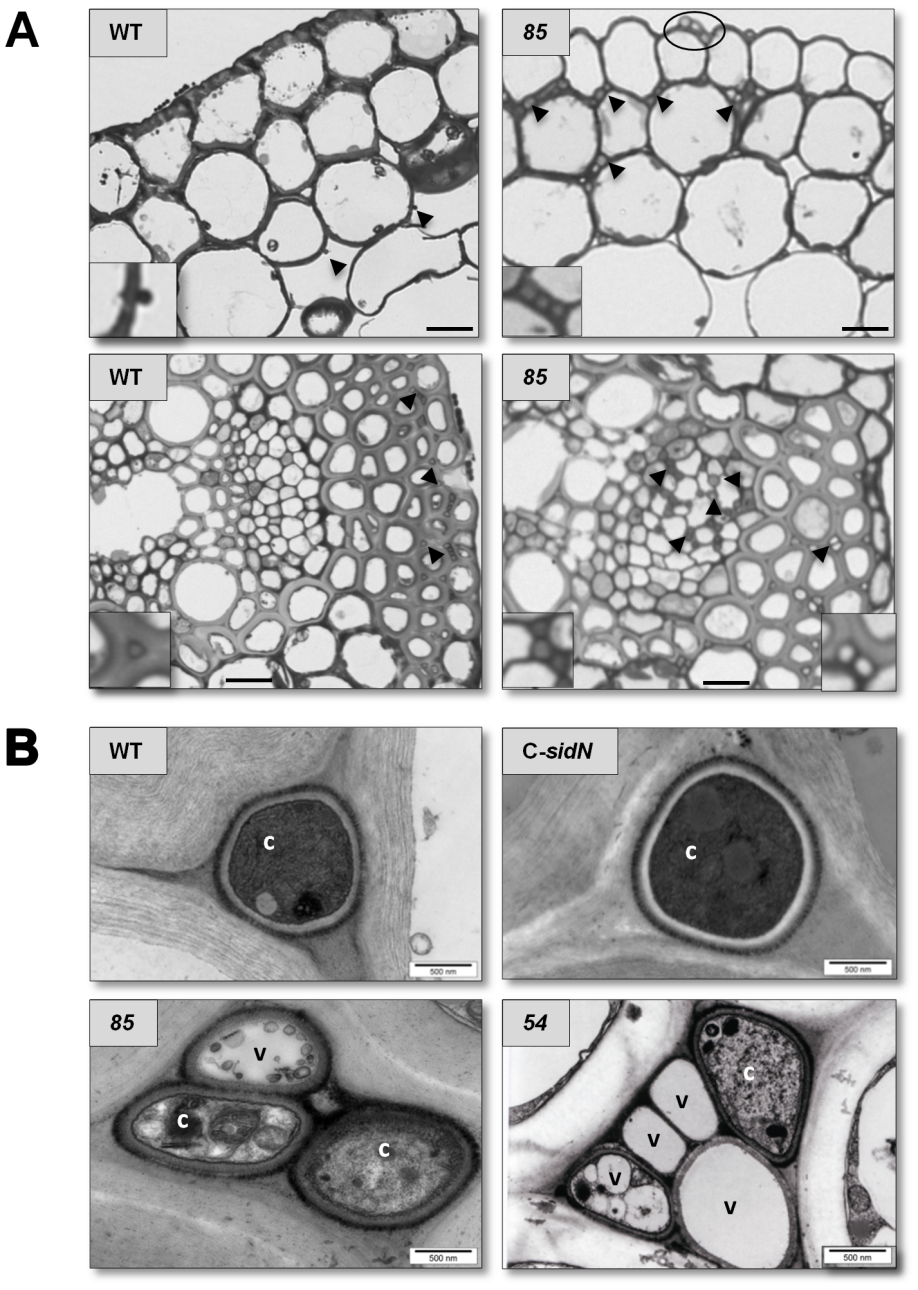
Abnormalities in the hyphal distribution and ultrastructure of *ΔsidN* mutants in perennial ryegrass plants. A. Light micrographs of 1 µM cross sections of the inner leaf sheath of perennial ryegrass infected with wild-type *E. festucae* Fl1 (WT) and *ΔsidN* mutant 85 (85) are shown. The top panel is a cross section of mesophyll cells, whereas the lower panel is a close up of vascular tissue. Representative hyphae are indicated by arrows and the circle on the 85 panel indicates epiphyllous hyphae. Inserts show higher magnification of the endophyte hyphae indicated by the arrowheads in the main panels. Bars = 20 µM. B. Transmission electron micrographs of cross sections of endophyte hyphae in the intercellular spaces of perennial ryegrass. Wild-type *E. festucae* Fl1 (WT), complemented *ΔsidN* strain (C-*sidN*) *ΔsidN* mutant 54 (54), *ΔsidN* mutant 85 (85) are shown. Samples shown were photographed from leaf sheath sections, with WT, C-*sidN*, and *ΔsidN* 85 hyphae located in mesophyll tissue, whereas *ΔsidN* 54 is in sclerenchma tissue. c, cytoplasm, v, vacuole. Bars = 500 nm.

Toluidine blue stained 1 µM cross sections of pseuodostems from WT, C-*sidN* and *ΔsidN* mutants 54 and 85 were examined to analyze the distribution of hyphae within different ages of leaf sheaths and blades (Figure 8A, B). We observed that hyphae from the WT or the complemented strains showed an even distribution of hyphae across all tissue types and tissue ages, but that hyphae from stunted plants infected with *ΔsidN* 54 or 85 were abnormally distributed. Specifically, the number of *ΔsidN* mutant hyphae were found to be the lowest in the inner developing leaf blade, and increased with age so that more hyphae were observed in the older outer leaf blade and sheaths (results not shown). We could also gain information about the cytoplasmic density of hyphae in specific tissues based on penetration of toluidine blue. Hyphae from the WT or complemented strain were generally dense in all tissues examined, but not so for the mutant strains. Dense hyphae were only observed in *ΔsidN* 54 or 85 infections from within the youngest tissue, the inner leaf blade, as well as hyphae located in the phloem of the vascular bundles. However, the majority of mutant hyphae located in the older leaf blade and all three sheaths were empty (Figure 8A shows hyphae located in the inner leaf sheath in both mesophyll and vascular tissue). Unlike the WT in which no hyphae were observed in the vasculature, mutant hyphae were found in large numbers in some of the vascular bundles (mostly in the phloem) of all of the tissue types examined (Figure 8A). Additionally, we noted many epiphyllous hyphae present with the mutant infection compared to few for the WT or complement, with most being large in diameter and apparently empty as hyphal contents were not stained at all with toluidine blue (Figure 8A). These light microscopy pictures indicate that the *ΔsidN* mutant is proliferating mostly in only the older parts of the plant, with typical WT-like growth confined to the inner leaf blade, where hyphae are few and stain densely. Generally, the densely-stained mutant hyphae were only seen in nutrient rich vasculature or in the inner leaf blade.

**Figure 9:**
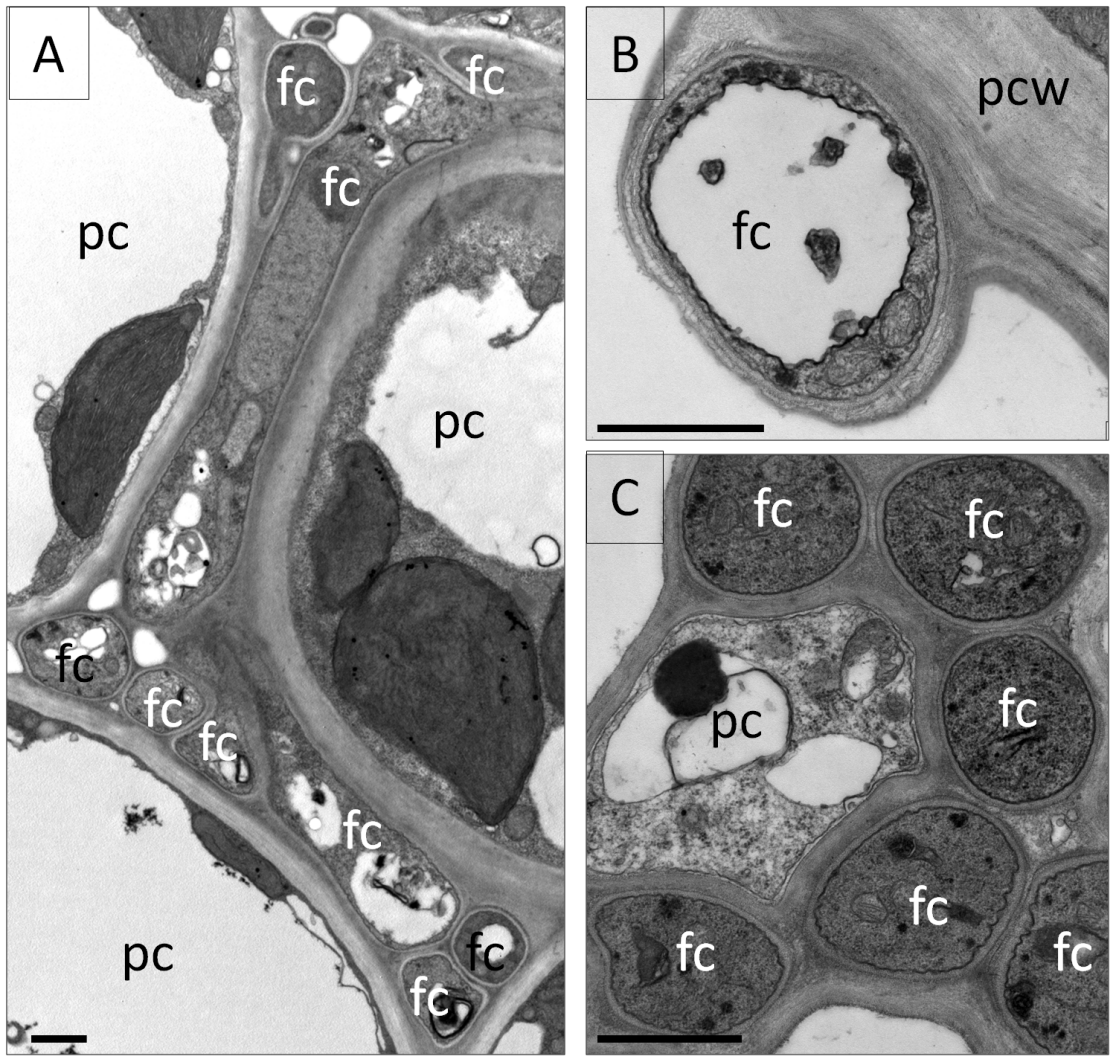
Ultrastructural features of the Δ*sidN* mutant - plant infection phenotype. **A.** Aberrant Δ*sidN 85* mutant hyphae in the mesophyll of the inner leaf sheath surrounding host cells. B. An epiphyllous Δ*sidN 85* mutant hypha on the outside of the outer leaf blade. C. Δ*sidN 85* mutant hyphae in the main vascular bundle of the inner leaf sheath. Bar indicates 1000 nm. fc indicates fungal cell, pc for plant cell, pcw for plant cell wall.

Transmission electron microscopy (TEM) of perennial ryegrass plants infected with WT, complement and *ΔsidN* mutants 54 and 85 confirmed the light microscopy findings (described above) and provided detailed images of various abnormalities apparent in the hyphal ultrastructure of *ΔsidN* mutants (Figure 8B). Frequently observed irregularities were atypical vacuolation as well as poorly stained hyphae versus rarely vacuolated and frequently densely-stained WT/complemented hyphae indicating that a characteristic feature of the hyphae of *ΔsidN* mutants is diffuse cytoplasm (Figure 8B, Figure 9). As already noted by light microscopy, hyphae located in the vasculature contained dense cytoplasm, and the contents as determined by TEM appeared very normal (WT-like) and hyphae were also mostly regularly shaped like WT (Figure 9). Additionally, we often observed asymmetrically shaped mutant hyphae of variable diameter in the mesophyll of various plant tissue types and occasionally, but only in mutant infected plants, the presence of clusters of hyphae orientated both longitudinally, obliquely and transversely surrounding host cells, but did not enter host cells (Figure 9). Epiphyllous hyphae were also able to be visualized by TEM in *ΔsidN* infections as they were so abundant, and were observed to always be highly vacuolated and encapsulated in plant host derived material (Figure 9).

Light microscopy and TEM observations showed that the *ΔsidN* mutants colonized the apoplastic space with multiple hyphae compared to single or few hyphae in the WT (Figures 8A, 8B, 9). This apparent increase in fungal biomass was quantified by real-time quantitative PCR (qPCR) of the single copy fungal gene, *NRPS1*, in total gDNA (plant and fungal; [60]). Relative to WT infected plant pseudostems (that comprise a mixture of enclosed immature emerging leaf blades and surrounding mature sheaths), the fungal biomass in plants infected with *ΔsidN* 54 was 1.4 fold higher, while in plants infected with *ΔsidN* 85 fungal biomass was 1.8 fold higher.

### Effects on the Chemical Phenotype of the Symbiotum

To determine whether the fungal extracellular siderophore migrated into the plant, we analyzed guttation fluid from perennial ryegrass infected with WT, *ΔsidN* 85 mutant and complemented C-*sidN* strains by targeted LCMS^n^, as we have found that this fluid provides a clean matrix for the detection of endophyte metabolites *in planta* [61]. While epichloënin A could not be detected *in planta*, ferriepichloënin A was detectable at trace levels in several samples from plants infected with the WT or C-*sidN* strains but could not be detected in plants infected with the *ΔsidN* 85 mutant (results not shown).

**Figure 10:**
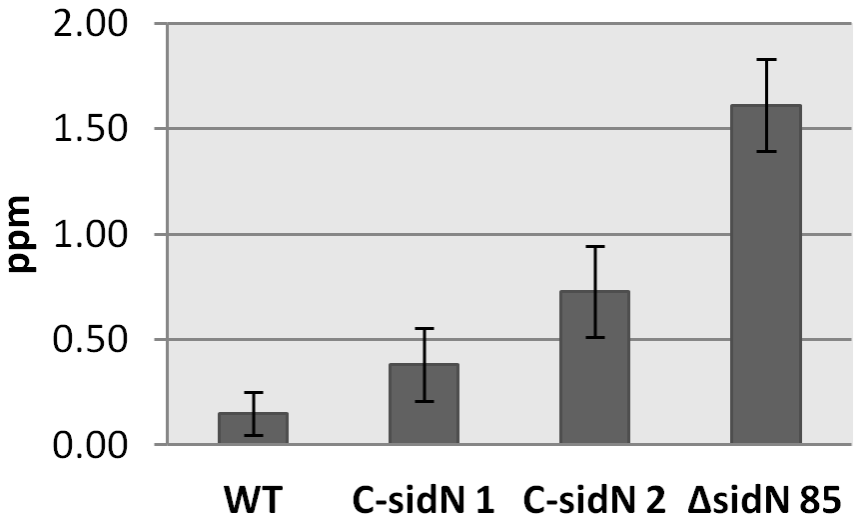
Elevated ergovaline levels detected in Δ*sidN* infected plants. HPLC analysis of ergovaline production was carried out on perennial ryegrass pseudostems infected with wild-type *E. festucae* Fl1 (WT), *ΔsidN* mutant 85 (*ΔsidN*), and complemented *ΔsidN* strains (C-*sidN* 1 and C*-sidN* 2). The numbers of plant reps used for analysis were 3–5 for each sample. Error bars indicate standard deviation.

Indirect effects of the *ΔsidN* 85 mutant on the chemical phenotype of the symbiotum were more marked. *E. festucae* strain Fl1, in association with perennial ryegrass, synthesizes three major classes of alkaloids: indolediterpenoids, of which lolitrem B is the major product; ergot alkaloids, of which ergovaline is the major product, and the pyrrolopyrazine peramine. Production of all three is minimal or undetectable in axenic cultures, but all are produced in significant amounts (of the order of µg/g) *in planta* [62]–[64]. Many biotic and abiotic factors influence the production of fungal alkaloids *in planta*, including mineral stress, for example nitrogen and phosphorous availability [60], [65], [66]. To test if the altered phenotype of *ΔsidN* 85 infected plants may have an effect on alkaloid production, we determined alkaloid concentrations in infected plants on at least 3 occasions. While considerable variation was observed in concentrations of lolitrem B, and to a lesser extent of peramine, ergovaline consistently accumulated to much higher levels in the *ΔsidN* 85 mutant plants compared to WT (10-fold for *ΔsidN* 85, Figure 10), a factor much larger than the 1.8-fold increase in fungal biomass in the identical plant tissue as described above. Other *ΔsidN* mutant strains showed a similar elevation in ergovaline levels (data not shown). In agreement with this, a transcriptomic experiment that compared plants either infected with WT or *ΔsidN* 54 strains by a custom designed Affymetrix GeneChip revealed that the expression of genes involved in the biosynthesis of ergovaline were highly elevated in the plants infected with the *ΔsidN* mutant (L. Johnson et al., unpublished results).

### Quantitative RT-PCR (RT-qPCR) Analysis of Iron-Regulated Genes

Siderophore mediated iron-uptake does not appear to be the only high affinity iron uptake system operational in *Epichloë* as a search of the *E. festucae* (E2368, Fl1) genome sequences revealed the presence of a putative ferroxidase, *fetC* and a putative high affinity iron permease, *ftrA* that form the bipartite Fet3/Ftr1 complex responsible for reductive iron assimilation (ferroxidation/permeation) [23] in these endophyte strains. Only one putative ortholog of *fetC* and *ftrA*, respectively, was identified. These two genes are co-localized in the genome and share a bidirectional promoter as is commonly found for other fungi [15], [16], [67]. Two other genes regulating iron homeostasis were also found in the genome; *sreA*, the GATA-type transcriptional repressor of iron uptake during iron-replete conditions [68], [69], and *hapX* involved in the repression of iron-utilizing proteins under iron deficiency mediated via the CCAAT-binding complex [70]–[73]. See Table S2 for detailed descriptions of identified genes used for RT-qPCR below.

**Figure 11:**
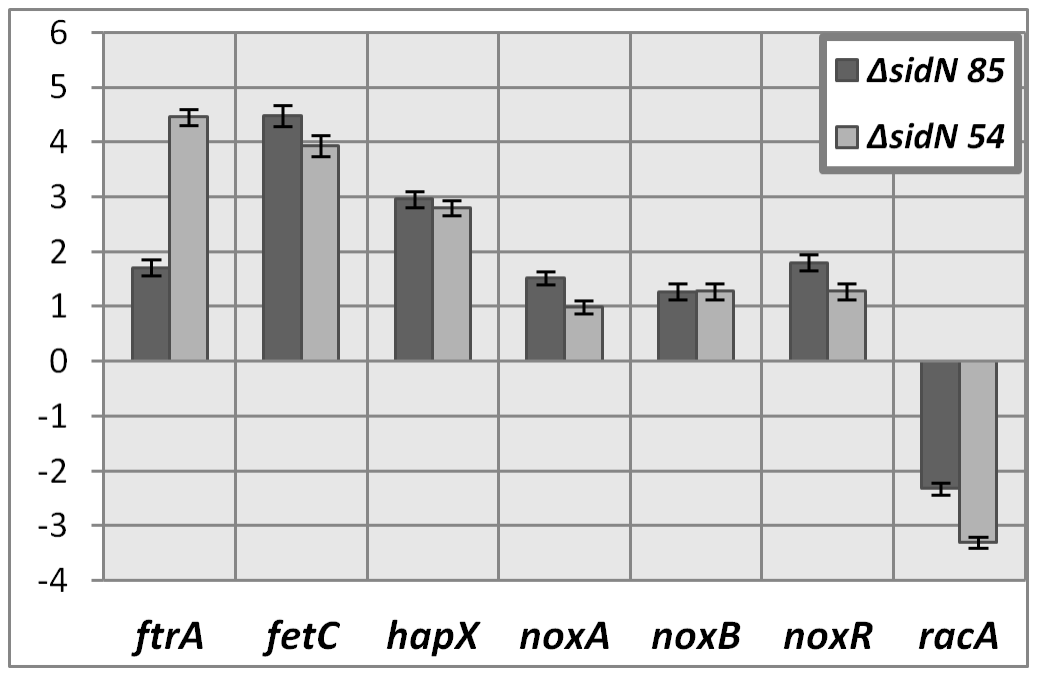
RT-qPCR of *E. festucae* iron-regulated genes and *Nox* genes in *ΔsidN* infected perennial ryegrass. Relative mean abundance relative to wild-type (fold difference displayed) of iron regulated gene expression (*ftrA*, *fetC*, *hapX*) and Nox gene expression (*noxA*, *noxB*, *noxR*, *racA*) in perennial ryegrass infected with Δ*sidN* mutants 54 and 85 are shown. For *ftrA*, *fetC*, *noxA* and *racA* results have a p-value of <0.001 and for *hapX*, *noxB* and *noxR*, the p-values are 0.002, 0.005 and 0.017 respectively. Error bars indicate SED.

To test our hypothesis that the Δ*sidN* mutants in perennial ryegrass, due to loss of their extracellular siderophore, are sensing changes in their cellular iron status, we quantified mRNA abundances of the putative *E. festucae* iron-repressed genes *fet3*, *ftr1*, and *hapX*. Significant up-regulation of all three iron-repressed genes in pseudostem tissue of Δ*sidN* 54 and 85 compared to WT infected plants was found, suggesting reduced intracellular iron levels in the Δ*sidN* mutants as a result of the loss of epichloënin A production (Figure 11).

### RT-qPCR Analysis of NADPH Oxidase (Nox) Genes

The roles of genes of the superoxide-generating NADPH oxidase (Nox) family have been well characterised in *E. festucae* and shown to be critical for symbiosis maintenance [74]–[77]. Perturbations in levels of reactive oxygen species (ROS) via mutation of components of the Nox complex in *E. festucae* affect regulation of hyphal growth and branching in the host and cause plant stunting. A number of shared phenotypic features of the *in planta* phenotypes of the Δ*sidN* mutants with the *E. festucae* Nox mutants, such as unrestricted hyphal growth and swollen hyphal tips, suggested that alterations in ROS could play a role in the Δ*sidN* mutant phenotype.

To investigate whether there were any alterations in the transcriptional regulation of the Nox complex, we quantified mRNA abundances by RT-qPCR of the Nox genes, *noxA* (required for symbiosis, [75]) and *noxB* (no known function, symbiosis not affected, [75]), as well as the regulators of this complex *noxR* [74] and the small GTPase *racA* (required for NoxA activation and regulated ROS production, [76]) in pseudostem tissue of plants either infected with WT or Δ*sidN* mutant strains. For perennial ryegrass plants infected with *ΔsidN* mutants, *noxA*, *noxB* and *noxR* genes were not differentially expressed relative to WT infected plants, but *racA* was down-regulated 2.2 to 3.3 fold in *ΔsidN* 85 and *ΔsidN* 54 infected plants respectively (Figure 11). To determine if altered *racA* expression resulted in a measureable difference in ROS production in the *ΔsidN* mutants, we examined ROS production in axenic cultures by both nitroblue tetrazolium (NTB) and DAB (3,3′-diaminobenzidine) staining for detection of superoxide and H_2_O_2_ respectively. However, the production of both superoxide and H_2_O_2_ of *ΔsidN* mutant colonies grown on iron-depleted (DM or DM with BPS) or iron-replete (DM supplemented with iron or PD) media showed no consistent significant difference compared to WT (results not shown). We did on the other hand observe an obvious increase in H_2_O_2_ (by DAB staining) across all mutant and control colony strains when grown on iron supplemented DM compared to iron-depleted DM alone (results not shown) indicating the general influence of free iron supplementation to increase ROS levels.

## Discussion

### *E. festucae sidN* Encodes the Novel Extracellular Siderophore Epichloënin A

This study presents the characterization of extracellular siderophore biosynthesis in the foliar grass endophytic fungus *E. festucae* of the Clavicipitaceae. Through targeted gene disruption of the NRPS gene *sidN* (formerly NRPS2) and complementation of a mutated Δ*sidN* strain, we confirmed that *sidN* encodes a siderophore synthetase required for the production of the novel extracellular siderophore epichloënin A, a variant of ferrirubin of the ferrichrome family. *In silico* analysis of the SidN domain architecture indicates that the protein consists of three complete A-T-C modules, two terminal T-C repeats and is identical to Type II ferrichrome NRPSs [57]. We can therefore infer that SidN activates three component amino acids required for the biosynthesis of a ferrichrome. This interpretation is consistent with the recent structural characterization of epichloënin A that showed it to be a cyclic octapeptide ferrichrome comprised of three contiguous units of *trans*-AMHO (the iron-chelating residues of hydroxamate siderophores [28], [57]), a glutamine and four glycine residues [53]. The third A domain (A3) of SidN has been experimentally confirmed to activate AMHO residues [52]. The other two A domains (A1 and A2) are therefore predicted to activate the two other remaining component amino acids [glutamine (novel component) and glycine (commonly incorporated)] of epichloënin A.

Investigations into the cellular location of epichloënin A and its ferri-form in the WT fungus grown on iron depleted defined medium (DM+BPS) showed that while epichloënin A was predominant in the culture supernatant, ferriepichloënin A was relatively more abundant in the mycelium. These results suggest epichloënin A produced by the fungus is secreted into the extracellular fluids where it binds available iron as ferriepichloënin A which is subsequently taken up by the fungus where the iron is retrieved (referred to as the “shuttle” mechanism; see Howard [78] for details on siderophore transport mechanisms). We are investigating whether ferriepichloënin A could also have an intracellular role, in addition to that of ferricrocin (putatively encoded by *NRPS9*), a well-known cellular siderophore which we have found only in mycelial extracts (L. Johnson et al., unpublished results).

### Loss of Epichloënin A Biosynthesis Adversely Affects the Mutualistic Perennial Ryegrass – Endophyte Association

Colonization of grass leaves by epichloae endophytes occurs by a unique process described in an intercalary growth model that has been experimentally verified by Christensen et al. [43]. The model illustrates how hyphae in the leaf extension zone, extend by intercalary growth (non-tip hyphal extension) at a rate that is synchronized with the expansion and migration of leaf cells. Once the leaf ceases to expand, hyphal growth also ceases, but hyphae remain metabolically active, continuing to be of benefit to their host [79]. This mode of endophytic growth *in planta* ensures that hyphal growth is synchronized with plant growth. The maintenance and regulation of this lifestyle is pivotal for the mutualistic nature of the associations studied here.

We have investigated the effects of loss of production of epichloënin A on the mutualistic association formed between *E. festucae* with its host grass perennial ryegrass and showed that loss of the extracellular siderophore is deleterious for the maintenance of mutualism. Plants infected with Δ*sidN* mutants were variably stunted, with altered tiller and root morphology compared to WT. The endophytic hyphae in Δ*sidN* plants are evidently no longer colonizing only by intercalary hyphal extension but also growing from hyphal tips in an unrestricted and sometimes disorientated manner with respect to the leaf axis, typified by hyper-branching and variable diameter. All of these effects are indicative of a change in the nature of the symbiotic relationship between the fungal endophyte and its host from being mainly mutualistic to detrimental. This study is the first account of the consequences of the loss of an extracellular siderophore in a mutualistic system and indicates that balancing of symbiotic iron homeostasis is an important factor in the maintenance of the mutualistic nature of grass-endophyte associations.

Despite the fact that loss of extracellular siderophores sometimes causes microbial pathogens to become less pathogenic, or a mutualistic symbiont to turn pathogenic-like, the commonality in this apparent paradox is that iron uptake appears obligatory for survival of all microbes, whether friend or foe. The symbiotic endophyte studied here is confined to the apoplastic spaces of above ground plant parts; it therefore depends completely on host iron for survival and our results suggest it must scavenge for iron via extracellular siderophore-mediated uptake. The inability of Δ*sidN* mutants to grow on iron depleted (DM+BPS) media indicates epichloënin A biosynthesis contributes significantly to iron assimilation in *E. festucae*. This is substantiated by the expression analysis of putative components of reductive iron assimilation (RIA) that are up-regulated in the Δ*sidN* infected plants, but appear unable to recompense for the lack of epichloënin A. RIA is therefore a functioning high affinity iron uptake system in *E. festucae* during *in planta* vegetative growth, but it does not appear to be the major system required for regulated iron uptake. Accordingly, up-regulation of *hapX*, the transcription factor that represses iron utilizing proteins in Δ*sidN* infected plants implies that the Δ*sidN* mutants require more iron than they are receiving via RIA alone. Another possible explanation is that RIA is bringing in sufficient iron but cellular iron handling processes have become deregulated due to the loss of epichloënin A, which could be acting as a regulatory iron sensor and/or cellular iron store (since epichloënin A is both secreted outside of the cell (iron-free) and also found intracellularly bound to iron), and consequently the incoming iron cannot be handled properly and thus utilized effectively. The observed proliferation of Δ*sidN* mutant hyphae *in planta*, in which the majority of hyphae (located in the older parts of the plant) are abnormal (frequently devoid of cytoplasm) and some appear dead (empty of cytoplasm), apart from those extensively colonizing the vasculature which have dense healthy looking cytoplasm suggests that hyphae are growing at a rate greater than can be sustained. The effect of the iron chelator BPS on the distribution of chitin in hyphae of the WT and mutant grown *in vitro* was also revealing in that it suggests that loss of RIA (via BPS amendment to the media) drastically changes chitin distribution and therefore cell polarity. We therefore conclude that changes in iron sensing and regulation are presumably the underlying agents responsible for the abnormal appearance of many mutant fungal hyphae *in planta*.

We observed an interesting increase on the endophyte *in planta* induced alkaloid ergovaline, in plants infected with Δ*sidN* mutant strains. This could be as a consequence of iron starvation or may be associated with the extensive hyphal tip growth and branching of fungal hyphae observed in the leaf sheaths of these plants. This pattern of growth is normally restricted to basal meristematic tissues, and a detailed dissection study of *N. lolii*-infected plants [64] found concentrations of ergovaline to be highly elevated in these tissues.

Oide et al. [19] present consequences of extracellular fungal siderophore loss in several fungal phytopathogens through *NPS6* gene deletion that resulted in reduced virulence and hypersensitivity to H_2_O_2_ [80], the latter being consistent with our findings of increased H_2_O_2_ sensitivity in Δ*sidN* mutants. The host penetration ability of these Δ*nps6* strains was not affected, however plant colonization was defective [19]. Application of iron restored this defect indicating that iron deficiency caused the lack of virulence in these fungi. Our work demonstrates that extracellular siderophores play a critical role in fungal-host relationships, but the consequences of loss of siderophore differ depending on the nature of that relationship (pathogenic vs. mutualistic).

### Symbiotic Iron Homeostasis, Oxidative Stress and ROS

The endophyte is housed in the apoplastic space where free iron is presumed to be a limiting factor. Our findings indicate that the endophyte requires its extracellular siderophore for mutualistic growth and supports the idea that iron is not readily accessible within the apoplast. The symbioses formed between epichloae endophytes and the Poaceae have co-evolved over evolutionary time, and maintaining symbiotic iron homeostasis is likely to be an intricately balanced process, and a key factor in keeping hyphal growth controlled. Iron homeostasis of the whole endophyte-grass symbiotum must be balanced for two primary reasons. Enough iron must be supplied for metabolism of both plant and fungal partners and excess must be avoided since free iron is toxic due to the formation of reactive oxygen species produced by the Fenton reaction giving rise to oxidative stress [4]. *In vitro* studies indicate the *ΔsidN* mutants are significantly more sensitive to H_2_O_2_ on iron-depleted medium than on iron-replete medium. This could simply be explained by a reduction in iron-dependent antioxidative enzymes, such as catalases and peroxidases, which require heme. A similar phenomenon was reported for Δ*nps6* strains where iron application enhanced tolerance to H_2_O_2_ and KO_2_ [19]. Intriguingly, the Fenton reaction does not appear to be the source of oxidative stress sensitivity since iron application would be expected to promote the Fenton reaction. Furthermore, Oide et al. [19] were able to show that both iron-saturated and nonsaturated siderophore (desferrioxamine) alleviated reactive oxygen species hypersensitivity indicating a possible protective role for siderophores against reactive oxygen species (ROS) *in vitro*; however their studies to demonstrate this role *in planta* were inconclusive. Further insights into the connections between ROS and iron regulation have been demonstrated from research into the monothiol glutaredoxins Grx3 and Grx4 of *Sacchromyces cerevisiae*, which show they function in the defense against oxidative stress through the regulation of iron homeostasis [81].

Research on the NADPH oxidase (Nox) complex in *E. festucae* associations with perennial ryegrass (through fungal mutants *NoxA*, *NoxB*, *NoxR*, *RacA* and *BemA*) have shown that Nox produced ROS plays a key role in regulating hyphal growth and branching in the host [74]–[77]. Disruption of the spatial and temporal production of fungal ROS leads to the loss of the characteristic features of endophyte-grass mutualistic associations. As the Δ*sidN* mutant infected plants shared some phenotypic features with the *E. festucae* Nox mutants, such as hyperbranching of hyphae within host leaves and plant stunting along with increases in tiller number, we explored whether the transcriptional regulation of the *E. festucae* Nox complex genes was affected in the Δ*sidN* infected plants. The Nox regulator *racA* was significantly down-regulated in both *ΔsidN* mutants studied, and although *noxR* gene expression was not differentially expressed in the *ΔsidN* mutants, NoxR requires a functional RacA to spatially regulate ROS production and control hyphal branching [74], [76]. However, this did not result in changes in ROS production levels in colonies of *ΔsidN* mutants grown on iron-depleted (or replete) medium. Based on these *in vitro* results, it seems less likely to find a significant difference in ROS production in the *ΔsidN* mutant infected host plants and was therefore not pursued.

Ultimately, to ensure continuance of mutualism, we postulate that maintenance of restricted hyphal growth of *E. festucae in planta* does not only require a functional Nox complex, but also the maintenance of iron homeostasis which is mediated via epichloënin A. To recapitulate, our results demonstrate that iron acquisition through siderophore-mediated iron uptake is necessary to maintain endophyte mutualism with perennial ryegrass.

## Methods

### Biological Materials and Growth Conditions

*Epichloë festucae* strain Fl1 (*ex cultivar SR3000*) and derivatives (this study) were grown on 2.4% potato dextrose agar (PDA, Difco Laboratories) and maintained as previously described [82], [83]. Defined medium (DM) for iron growth studies and chemical analyses were modified from Mantle and Nisbet [84], with yeast extract replaced with 0.6 µM thymine and iron was omitted. DM medium was also supplemented with the following as indicated: 100 µM BPS (bathophenanthrolinedisulfonic acid; Sigma), 20 µM FeSO_4_, 20 µM FeCl_3_.

### Plant Inoculations and Growth Conditions

Inoculation of seedlings of perennial ryegrass ‘Nui’ was performed using the method of Latch and Christensen [85]. Plant growth from seed to 6 weeks-old is as described by Tanaka et al. [86], except the potting mix was of the following composition: 60% peat, 40% coarse sand with slow release fertiliser (nutricote with F.T.E.) and dolomite lime at 2.4 kg/m. Determination of endophyte infection was performed by immunoblotting [87]–[89] and light microscopy of epidermal leaf sheaths by aniline blue staining [89].

### Examination of Asexual Sporulation *In Vitro*

Plates containing 2% water agar were inoculated with small agar plugs of mycelium and grown for 2 weeks at 22°C, followed by 2 weeks at 4°C. Slides were then mounted with agar blocks using a stereomicroscope to locate appropriate regions for counting of conidia by light microscopy using bright field optics. To determine conidia number from each colony mounted, 5 regions were counted starting from the colony edge and moving towards the colony centre. Conidia numbers were recorded and the data graphed is the mean spore count obtained from three independent colonies and three technical replicates.

### Oxidative Stress Test

Sensitivity to oxidative stress was examined using a final concentration of 0.7 mM H_2_O_2_ in the medium (DM or PD) and tests were repeated at least 3 times. The colony diameters obtained from growth on DM or PD with or without H_2_O_2_ supplementation were measured and recorded at 7 days (at 22°C). The ratios of DM/DM+H_2_O_2_ or PD/PD+H_2_O_2_ were recorded for each fungal strain. Data were analysed by an analysis of variance (ANOVA) and the least significant difference used to compare stress sensitivity of strains.

### DNA and Fosmid Library Preparations and Standard Molecular Techniques

Plasmid or fosmid DNA was isolated using the QIAprep Spin Miniprep Kit (Qiagen). Fosmid DNA prior to extraction was induced to high copy number using the manufacture's protocol (Epicentre Biotechnologies). *Escherichia coli* TOPO strain (Invitrogen) was used to propagate plasmids using standard techniques [90].

Fungal genomic DNA (gDNA) for Southern Blot analysis was isolated using freeze-dried or fresh mycelium by the method of Yoder [91]. For identifying a positive disruption event by PCR, a small scale DNA protocol was used. Transformants were inoculated from one small mycelial plug (taken from the outer colony margin) into 100 µl of PD broth in a 1.5 ml tube and grown at 26°C for 3 days. Supernatant was removed and freeze-dried mycelia were ground and extracted in 150 µl of lysis buffer (100 mM Tris-Cl, pH 8.0, 100 mM EDTA and 1% SDS). Following incubation at 70°C for 30 min, the lysates were mixed with 150 µl of 5 M potassium acetate solution and incubated on ice for 10 min. After centrifugation for 10 min, the supernatant containing the fungal DNA was precipitated with 0.7 volumes of isopropanol. The DNA pellet was washed once with 70% ethanol and finally dissolved in 20 µl of water. 1 µl of DNA was used for PCR analysis. Isolation of high molecular weight DNA for fosmid library preparation from protoplasts was performed based on the method by Denning et al. [92] and modified as follows: 500 µl of protoplasts in STC buffer were prepared as described by Young et al. [93], [94], lysed, and then proteinase K treated and phenol-chloroform purified as stated by Denning et al. [92]. The resulting aqueous phase was precipitated with isopropanol, followed by RNase A treatment (0.06 mg of RNase A (Invitrogen); incubation at 37°C for 30 min), then precipitated with 100% ethanol, and the resulting pellet washed with 70% ethanol, air dried and finally resuspended in water.

For Southern blot analysis, restriction enzyme digested gDNA was transferred to Hybond N+ (Amersham Pharmacia Biotech) overnight with 0.4 M NaOH. Filters were hybridized at 42°C with digoxigenin (DIG)-labelled DNA probes which were labelled by PCR incorporation of DIG-11-dUTP according to the manufacturer's instructions (Roche).

Standard PCR conditions for amplification from DNA templates were performed in a 15 µl reaction volume containing 20 mM Tris-HCl (pH 8.0), 50 mM KCl, 1 mM MgCl_2_, 200 µM dNTPS, 0.3 µM of each primer, 0.9 U of *Taq* DNA polymerase (Invitrogen). Cycling program (for products less than 2.0 kb) was as follows: one cycle at 95°C for 2 mins; 30 cycles at 95°C for 30 s, 58°C (dependant on primer TM) for 30 s and 72°C for 30 s; 72°C for 10 min.

An *E. festucae* fosmid library using gDNA isolated from protoplasts of *E. festucae* strain FL1 was constructed using the CopyControl Fosmid Production Kit (Epicentre Biotechnologies) according to the manufacturer's instructions. Broth cultures from 3840 independent colonies were obtained and a single pooled 384-well plate (containing 3840 colonies) was screened by PCR using primers Sid1F & Sid1R. See Table S1 for details of primers used in this study. Standard PCR conditions were used as cited above.

### Generation of Disruption and Complementation Vectors, *E. festucae* Transformation and Characterization of Transformants

We had previously probed a *N. lolii* Lp19 lambda library with a *NRPS2* derived PCR product, and identified one lambda clone of approximately 5.8 kb with the following partial domain structure: A (truncated)-T-C-A-T-C-T-C-T-C. This information was used for performing a gene disruption by homologous recombination in the sexual relative, *E. festucae* wild-type (WT) strain Fl1. The gene disruption vector was constructed using Multisite Gateway Three-fragment Vector Construction Kit (Invitrogen) so that a portion of the genomic region encoding the third A domain of *sidN* was replaced with the hygromycin B resistance gene (see Figure S3A). The 5′ and 3′ entry clones were respectively PCR amplified from *E. festucae* (primer pairs Sid5F and Sid5R/Sid6F and Sid6R), then combined to produce a destination vector containing inserts of approximately 3.0 kb of 5′ NRPS coding sequence, followed by a 4.0 kb hygromycin B resistant gene (originally amplified from pAN7-1) and approximately 3.0 kb of consecutive 3′ NRPS sequence after a deleted region of 35 bp.

Protoplasts were prepared as described by Young et al. [93], [94]. PEG-mediated transformation of protoplasts was carried out with a linear PCR product of ∼10 kb derived from the *sidN* gene disruption construct and obtained by PCR using the *ΔsidN* disruption vector as a template with primers sid2F and sid2R. PCR amplification in a 50 µl reaction volume contained 1× Tuning buffer with 5 mM Mg^2+^ (Eppendorf), 500 µM dNTPs, 400 nM of each primer, 1 ng of plasmid DNA and 2 units of TripleMaster Polymerase Mix (Eppendorf). Cycling program was as follows: one cycle at 93°C for 3 min; 20 cycles at 95°C for 15 s, 56–58°C for 30 s, 68°C for 8 min; 68°C for 10 min.

Transformants generated from the gene disruption experiment were initially screened by PCR with primers specific to *sidN* (Sid4F and Sid4R) which flank the hygromycin B resistance gene (wild-type 0.24 kb, disruption 4.24 kb; data not shown). Southern blot analysis using a probe (amplified with primers Sid2R and Sid3F) specific to *sidN* which also spans the hygromycin B resistance gene in the disruption construct confirmed the disruption event (mutant strains showed loss of WT 5.45 kb band and a new band of 9.45 kb) (see Figure S3B). Putative *ΔsidN* mutants were subsequently confirmed by southern blotting to have the disruption event (see Figure S3B).

Complementation of Δ*sidN* 85 strain was carried out by co-transforming 5 µg of fosmid DNA [containing the entire promoter and open reading frame of *sidN* (as well as 2 additional ORFs located 3′ of *sidN*)] along with 1 µg of circular PII99 [95] carrying the selectable antibiotic resistant marker geneticin.

The transformation methodology of Vollmer and Yanofsky [96] with modifications by Itoh et al. [97] was used for gene disruption and complementation experiments. Disruption transformants were selected on regeneration (RG) medium containing hygromycin (150 µg ml^−1^), whereas geneticin resistant transformants derived from the complementation experiment were selected on RG medium containing 200 µg ml^−1^ of geneticin. Mycelium was subcultured three times for nuclear purification of transformants.

Complementation transformants were identified by their ability to grow on iron-depleted medium containing the iron chelator BPS. Confirmation was obtained by assaying culture filtrates from a subset of positive transformants for siderophore production by LCMSMS.

### Determination of Fungal Concentration *in planta* by qPCR

qPCR on gDNA isolated from endophyte-infected plants (pseudostem) was performed on a MyiQ cycler (Bio-Rad) using primers designed to a nonribosomal peptide synthetase (*NRPS-1*) gene as described in Rasmussen et al. [60]. Concentration of endophyte in infected tissues is expressed as the number of copies of the single copy *NRPS1* gene per total gDNA (from plant and fungus).

### RNA Preparation and Expression Analysis

Total RNA was extracted from frozen fungal mycelium using TRIzol reagent (Invitrogen). For extraction from perennial ryegrass tissues, RNA was at first isolated using TRIzol and further purified through the Plant RNeasy Kit (Qiagen) as follows: ∼30 mg of frozen plant tissue was ground to a fine powder, mixed well with 750 µl of TRIzol and incubated at room temperature for 5 min. Following centrifugation at 12K rpm for 10 min, the supernatant was transferred to a new tube and 150 µl of chloroform added. After mixing well for 15 s and incubating at room temperature for 3 min, samples were centrifuged at 13.2 K rpm for 5 min. The aqueous phase was transferred to a fresh tube and an equal volume of 70% ethanol added before loading onto a Qiagen RNeasy mini column (pink). From this point onwards, total RNA was column purified using the manufacturer's instructions (Qiagen). The optional DNase digestion on the column step was also carried out for plant samples.

Any DNA still remaining was removed from total RNA by treating 10 µg of RNA with 20 U of DNase I, RNase-free (Roche) and 5 mM MgSO_4_ at 37°C for 30 min, followed by 5 min at 75°C. First strand cDNA primed with oligo(dT) was synthesized using the ThermoScript RT-PCR system (Invitrogen) from 1 or 2 µg of denatured total RNA by incubation at 50°C for 60 min, followed by 85°C for 5 min.

For RT-PCR expression analysis of *sidN* from axenic liquid DM cultures, the primer pair Sid4F and Sid4R was used to amplify cDNA using standard PCR conditions. Primers to the *Neoptyphodium lolii* actin gene (Acting and ActinR) were used to check for cDNA quality.

Two-step RT-qPCR analysis of perennial ryegrass tissue was performed on an iCycler (MyiQ Single Color Real-Time PCR Detection System, Bio-Rad) using Power SYBR Green PCR Master Mix (AB applied biosystems). The following cycle parameters were applied: 95°C for 5 min and then 40 cycles of 95°C for 20 s, 54–56°C for 20 s, and 72°C for 30 s followed by a melt curve analysis. Primer efficiency ranged between 94% and 110%. Primers for RT-qPCR (see Table S1) of putative iron-regulated genes, *Ftr1*, *Fet3* and *HapX* were designed to sequences from the *E. festucae* genome strain E2368. These genes were identified by performing tblastx queries of the *E. festucae* (E2368) genome with characterized iron-regulated genes from other fungal species (http://csbio-l.csr.uky.edu/ef2011/blast/blast.html). The deduced amino acid sequences of the identified genes from *E. festucae* (strain E2368) were then compared to the NCBI protein database by blastx (see Table S2 for descriptions of identified genes used for RT-qPCR).

Primers for the RT-qPCR analysis of NADPH Oxidase (Nox) genes (see Table S1) were designed to *E. festucae* sequences sourced from accession numbers: AB236860 (*noxA*), AB236861 (*noxB*), AB260938 (*noxR*) and AB260937 (*racA*). Purified PCR products were used to create a calibration curve for each gene-specific primer pair. Two endophyte housekeeping genes (a 60S ribosomal protein L35 and gamma actin, see Table S1 for details) were used to normalize the expression levels of the different genes. An analysis of variance (ANOVA) was performed from the geometric mean obtained from three biological replicates and two or three technical replicates for each primer pair. The least significant difference was used to compare the samples. P-values obtained for each transcript were: *ftrA* P<0.001; *fetC*, P<0.001; *hapX* P<0.002, *noxA* P<0.001, *noxB* P = 0.005, *noxR* P = 0.017; *racA* P<0.001.

### DNA Sequencing and Bioinformatics

Sequencing was performed with the Big-Dye (Version 3) chemistry (*PE biosystems*) and the products separated on an ABI Prism 3100 automated sequencer (Applied Biosystems).

Sequence comparisons were performed against local mirrors of a number of public databases, including GenBank, RefSeq, Cogeme and a selection of fungal genomes from the Broad Institute. Algorithms tblastx, blastx and blastn were employed to generate alignments [98]. Contigs were assembled using Vector NTI Advance 9.1.0, ContigExpress (Invitrogen) and SEQUENCER 4.6 (Gene Codes Corporation). NRPS domain structure was determined using a local mirror of InterProScan combined with a manual annotation based on the identification of motifs within domains as described by Schwarzer et al. [99].

### Analysis of Siderophores in Culture by LCMS

Samples of supernatant from liquid cultures grown for 2 weeks were separated for analysis by centrifugation. The residual mycelium was freeze-dried and finely ground, and extracted with water, and siderophores separated by solid phase extraction [100]. All samples were stored at −20°C prior to analysis. Milli-Q water and HPLC grade solvents were used for LCMS. The samples were thawed prior to analysis and transferred to a HPLC vial with 200 µl insert. Samples were kept at 5°C in the autosampler, and 10 µl subsamples were injected. Analytes were eluted through a C18 Luna column (Phenomenex Torrence, CA, USA) (150×2 mm, 5 µm) at a flow rate of 200 µl min^−1^ using a Thermo Finnigan Surveyor HPLC system with a solvent gradient (solvent A: H_2_O 0.1% formic acid; B: MeCN 0.1% formic acid), starting with 5% B, 95% A for 5 min and then increasing to 33% B after 15 min, then to 95% B by 20 min where the composition was held for 5 min to wash the column before being returned to 5% B to re-equilibrate the column. Mass spectra were detected with a linear ion trap mass spectrometer (Thermo LTQ) using ESI in positive ion mode. The spray voltage was 4.5 kV and the capillary temperature 275°C. The flow rates of nitrogen sheath gas, auxiliary gas, and sweep gas were set to 20, 5, and 10 (arbitrary units), respectively. Ferriepichloënin A was detected as an MS^1^ ion of *m/z* 569 [MH_2_]^2+^; epichloënin A was detected as an MS^1^ ion of *m/z* 542 [MH_2_]^2+^. *Cis*-AMHO in a standard solution (kindly provided by T. Verne Lee, University of Auckland, New Zealand) and *trans*-AMHO in culture supernatants were detected by monitoring the MS^1^ ion of *m/z* 261, and MS^2^ spectra were recorded by selecting and fragmenting this ion.

### Analysis of Siderophores in Guttation Fluid by LCMSMS

Guttation fluid was collected from the association of *L. perenne* G1057 with *E. festucae* Fl1, as previously described by Koulman et al. [61]. In brief, plants were placed overnight in a closed container and in the early morning the fluid accumulated at the leaf ends of a plant was collected with a glass pipette, transferred to a plastic container, and stored at −20°C until analysis.

Samples were analysed by direct injection of a 10 µl subsample of guttation fluid into a Thermo Finnigan Surveyor HPLC system attached to a linear ion trap mass spectrometer (Thermo LTQ) operated as described above. Ferriepichloënin A was detected by selecting and fragmenting the parent [MH2]^2+^ ion *m/z* 569±2 (35% relative collision energy), and selecting and fragmenting the product ion *m/z* 1024±2 (35% relative collision energy) and monitoring the total ion current.

### Alkaloid Analysis

HPLC was used to measure *in planta* levels of lolitrem B, ergovaline and peramine alkaloids as previously described [101], [102].

### Light and Transmission Electron Micrographs

Hyphae within leaf sheaths were examined by light microscopy of epidermal leaf sheaths by aniline blue staining [89].

For calcofluor (1 µg/ml) staining, mycelia were grown on microscopy slides covered with a thin layer of DM medium. Samples were directly incubated with the dye for up to 20 minutes at room temperature and washed with water to remove background fluorescence.

Superoxide production was detected by NTB staining using the method described by Takemoto et al. [74] except slides were overlaid with DM or PD media.

H_2_O_2_ production was examined by DAB staining (DAB forms a brick-red precipitate upon reaction with H_2_O_2_ [103] of cultures grown on DM and PD media as described by [104].

Examination of sections from pseudostem samples (approximately 1 mm long) required fixation in 3% glutaraldehyde and 2% formaldehyde in 0.1 M phosphate buffer pH 7.2 for 2 h at room temperature, followed by treatment in 1% osmium tetroxide in 0.1 M phosphate buffer pH 7.2 for 0.5 h at room temperature. The tissues were then washed 3 times in 0.1 M phosphate buffer pH 7.2, dehydrated in an acetone/water series and two times in 100% acetone. Samples were infiltrated with an acetone/polarbed 812 resin mixture (50/50, v/v), and then embedded in fresh resin mixture in silicone rubber moulds and cured for 48 h at 60°C. To examine the distribution of hyphae within different ages of leaf sheaths, cross sections (approximately 1 µm thick) were prepared from the treated pseudostem samples, and stained with toluidine blue for examination by bright field light microscopy. For examination with a Philips 201C transmission electron microscope, ultra-thin sections were cut and stained with saturated uranyl acetate in 50% ethanol for 4 min followed by lead citrate for 4 min.

### Accession Numbers

Sequence data from this article can be found in the GenBank/EMBL databases under the following accession numbers: *N. lolii NRPS2* (EF19536), *N. lolii* (strain Lp19) *sidN* (JN132404), *E. festucae* (strain Fl1) *sidN* (JN132407), *E. festucae* (strain E2368) *sidN* (JN132403), *E. festucae* (strain E2368) *ftrA* (JN132405), *E. festucae* (strain E2368) *fetC* (JN132406), *E. festucae* (strain E2368) *hapX* (JN132401), *E. festucae* (strain E2368) *actG* (FJ826616.1), *E. festucae* (strain E2368) *actA* (FJ379533.1), *N. lolii* RP(L35) (JN132402).

## Supporting Information

Figure S1 **Δ** ***sidN*** **Mutants Accumulate the Epichloënin A Precursor** ***Trans*** **-AMHO.** A. Positive electrospray LS-MS extracted MS^1^ ion chromatograms of the parent [M+H]^+^ion (*m/z* 261) and CID MS^2^ spectra of authentic *cis*-AMHO standard and putative *trans*-AMHO from Δ*sidN* 85 mutant. B. Relative concentrations of *trans*-AMHO in extracts of mycelium from cultures of wild-type *E. festucae* Fl1 (WT), *ΔsidN* mutant 85 (*ΔsidN*), and a complemented *ΔsidN* strain (C-*sidN*) grown under Fe-depleted conditions detected by LCMS (MS^1^ *m/z* 261) (arbitrary units).
(TIF)

Figure S2 **Conidiation in** ***E. festucae*** **is Reduced by Loss of Epichloënin A.** A. Spore counts produced from colonies of *E. festucae* Fl1 (WT), complement (C-sidN) and Δ*sidN* mutant 85 (Δ*sidN*) grown on water-agar for 2 weeks at 22°C, followed by 2 weeks at 4°C. Data were generated from three independent colonies and three technical replicates. Error bars = standard error. B. Microscopic examination of WT and Δ*sidN* water-agar colonies used for spore counts showing spores on coil structures and hyphal strands
(TIF)

Figure S3 **Gene Disruption Strategy and Southern Blot Analysis.** A. A linear *sidN* disruption vector is shown annotated with corresponding modular NRPS domain structure [A-domain (adenylation), T-domain (peptidyl carrier) and C-domain (condensation)] and restriction enzyme sites for *Hin*dIII (H). The hygromycin (HYG) cassette is indicated to insert into the A-domain of SidN (which was deduced from partial *sidN* sequence). B. An autoradiograph of a DNA gel blot of *Hin*dIII digested genomic DNA of Δ*sidN* mutants 54, 82 and 85 along with wild-type (WT) and ectopic (E) controls probed with a DIG-labeled *sidN* PCR product amplified with primers Sid3F and Sid2R.
(TIF)

Table S1 Detailed Information of the Primers Used in This Study.
(DOCX)

Table S2 Iron-Responsive Genes in *E. festucae*.
(DOCX)
