# Supplementary figures and images for: An Extracellular Siderophore Is Required to Maintain the Mutualistic Interaction of Epichloë festucae with Lolium perenne
Source: PLoS Pathog. 2013 May 2;9(5):e1003332. doi: 10.1371/journal.ppat.1003332 (PMC3642064; doi:10.1371/journal.ppat.1003332)

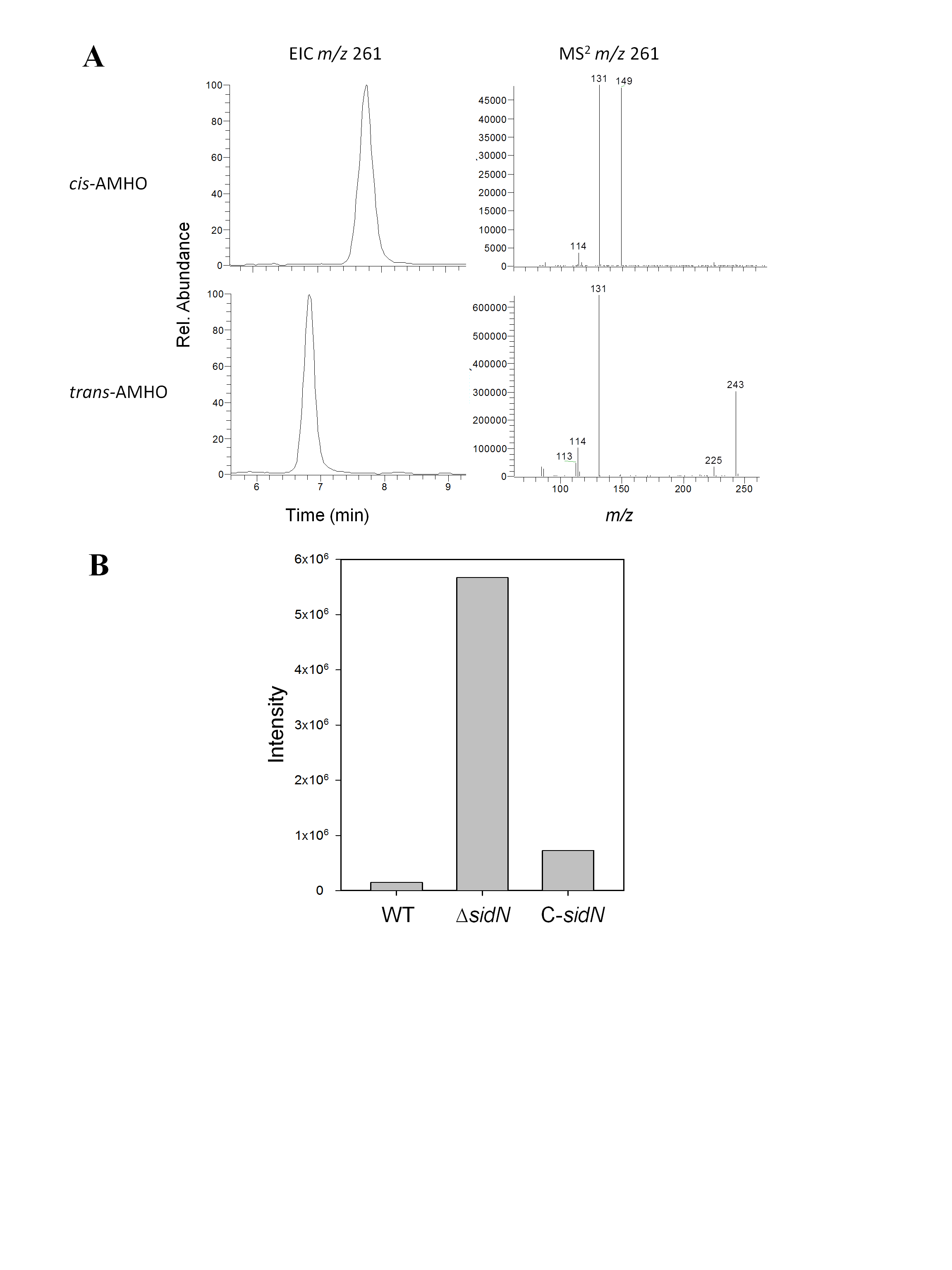

Supplement: Figure S1 — Δ sidN Mutants Accumulate the Epichloënin A Precursor Trans -AMHO. A. Positive electrospray LS-MS extracted MS1 ion chromatograms of the parent [M+H]+ion (m/z 261) and CID MS2 spectra of authentic cis-AMHO standard and putative trans-AMHO from ΔsidN 85 mutant. B. Relative concentrations of trans-AMHO in extracts of mycelium from cultures of wild-type E. festucae Fl1 (WT), ΔsidN mutant 85 (ΔsidN), and a complemented ΔsidN strain (C-sidN) grown under Fe-depleted conditions detected by LCMS (MS1 m/z 261) (arbitrary units). (TIF) [file ppat.1003332.s001.tif]

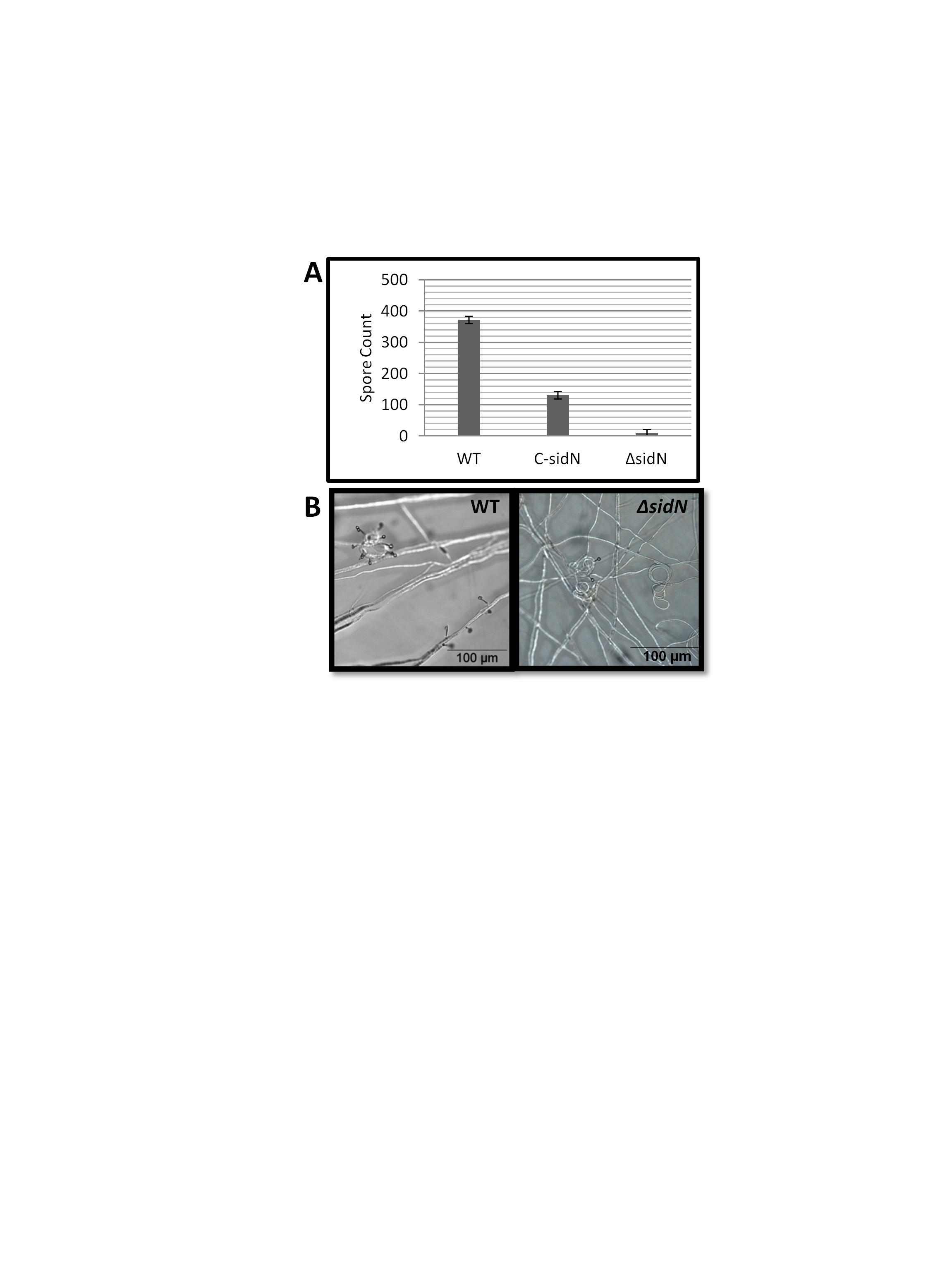

Supplement: Figure S2 — Conidiation in E. festucae is Reduced by Loss of Epichloënin A. A. Spore counts produced from colonies of E. festucae Fl1 (WT), complement (C-sidN) and ΔsidN mutant 85 (ΔsidN) grown on water-agar for 2 weeks at 22°C, followed by 2 weeks at 4°C. Data were generated from three independent colonies and three technical replicates. Error bars = standard error. B. Microscopic examination of WT and ΔsidN water-agar colonies used for spore counts showing spores on coil structures and hyphal strands (TIF) [file ppat.1003332.s002.tif]
