# Supplementary material for: An Extracellular Siderophore Is Required to Maintain the Mutualistic Interaction of Epichloë festucae with Lolium perenne
Source: PLoS Pathog. 2013 May 2;9(5):e1003332. doi: 10.1371/journal.ppat.1003332 (PMC3642064; doi:10.1371/journal.ppat.1003332)
Supplement: Table S1 — Detailed Information of the Primers Used in This Study. (DOCX) [file ppat.1003332.s004.docx]

**Supplemental Table 1.** Detailed Information of the Primers Used in This Study.

| **Primer name** | **Gene name** | **Description** | **Sequence** | **Purpose** |
| --- | --- | --- | --- | --- |
| Sid1F | *sidN* | NRPS: Epichloënin A | CTCTTCAACGCCCACTTGAATA | Fosmid screening |
| Sid1R | *sidN* | NRPS: Epichloënin A | CTCTGCTTCCAACAAGCCGTG | Fosmid screening |
| Sid2F | *sidN* | NRPS: Epichloënin A | GCCAGACGTTTTCATGCCAT | Vector PCR |
| Sid2R | *sidN* | NRPS: Epichloënin A | TGGTCTATGCTCGTTCCTTG | DNA gel blot & vector PCR |
| Sid3F | *sidN* | NRPS: Epichloënin A | GCAAATTCTCACCGTGGTCA | DNA gel blot |
| Sid4F | *sidN* | NRPS: Epichloënin A | ACGGAGGTTACGATTGGATGCA | Transformant screening & *sidN* RT-PCR |
| Sid4R | *sidN* | NRPS: Epichloënin A | TCGCCGGTCTTATACATCTTTC | Transformant screening and *sidN* RT-PCR |
| Sid5F | *sidN-*  entry clone | NRPS: Epichloënin A  & entry clone | GGGGACAACTTTGTATAATAAAG  TTGCACGCTGACCGCTGAGCTTCC | Entry clones |
| Sid5R | *sidN-*  entry clone | NRPS: Epichloënin A  & entry clone | GGGGACAGCTTTCTTGTACAAAG  TGGGTCGGAGTCTGGCAAGATTC | Entry clones |
| Sid6F | *sidN-*  entry clone | NRPS: Epichloënin A  & entry clone | GGGGACTGCTTTTTTGTACAAAC  TTGTTGCTCACGTTGCTGGCTCCTG | Entry clones |
| Sid6R | *sidN-*  entry clone | NRPS: Epichloënin A  & entry clone | GGGGACAACTTTGTATAGAAAA  GTTGTCTAACCTGGTCTATGCTCG | Entry clones |
| Actin_F | *actA* | *N. lolli* actin | AAGAAGTTGCCGCTCTCGTCATC | *sidN* RT-PCR |
| Actin_R | *actA* | *N. lolli* actin | CGGGGACGGCCGACGATGGAG | *sidN* RT-PCR |
| noxA_F | *noxA* | NADPH oxidase A | CCACGATGAGAGATGGTATTC | q-RT-PCR |
| noxA_R | *noxA* | NADPH oxidase A | GCTACAGAGGGTCCACAGAAAT | q-RT-PCR |
| noxB_F | *noxB* | NADPH oxidase B | CGCAGACAAAGACACCATCA | q-RT-PCR |
| noxB_R | *noxB* | NADPH oxidase B | AAGAAGACACCCGCCTCAC | q-RT-PCR |
| noxR_F | *noxR* | NADPH oxidase R | AAGATGTTGGTATGCAGGATTT | q-RT-PCR |
| noxR_R | *noxR* | NADPH oxidase R | GTGTAACCCTCGGCCTCCT | q-RT-PCR |
| racA_F | *racA* | small GTPase | CCCAACATTCCCATCATCTT | q-RT-PCR |
| racA_R | *racA* | small GTPase | GCTCCATTCGCTTTTGTCTC | q-RT-PCR |
| ftrA_R | *ftrA* | high-affinity iron permease | CTTGGTCTGCATGATTGTCG | q-RT-PCR |
| ftrA_F | *ftrA* | high-affinity iron permease | TAATTCTCGCTGTTCTCCCA | q-RT-PCR |
| fetC_F | *fetC* | ferrioxidase | GTCATCTCGGGGCTTGTC | q-RT-PCR |
| fetC_R | *fetC* | ferrioxidase | AGGGTCGGGTGGTGTATTTT | q-RT-PCR |
| hapX_F | *hapX* | bZIP-type regulator | CCCAATCCTCCCATCAAAC | q-RT-PCR |
| hapX_R | *hapX* | bZIP-type regulator | TCCATCAGCAGTCATCTCCA | q-RT-PCR |
| 174_F | RP(L35) | *N. lolli* 60S ribosomal protein L35 | CTCTCGACCTCCGTGTCAAG | q-RT-PCR |
| 174_R | RP(L35) | *N. lolli* 60S ribosomal protein L35 | GAAGTGAGTGGTGCGCTTC | q-RT-PCR |
| 178_F | *actG* | *E. festucae* strain E2368 gamma-actin | AAGTGTGATGTCGATGTCCG | q-RT-PCR |
| 178_R | *actG* | *E. festucae* strain E2368 gamma-actin | TGCATACGGTCGGAGAGAC | q-RT-PCR |

**Supplemental Table 2.** Iron-Responsive Genes in *E. festucae*

| Gene Name | Putative function | Closest characterised  BLAST hit | Percentage  identity* | References |
| --- | --- | --- | --- | --- |
| *ftrA*  *fetC*  *hapX* | high-affinity iron permease  ferrioxidase  bZIP-type regulator | *Aspergillus fumigatus* AAT84596  *Gibberella zeae* XP_385335  *Aspergillus fumigatus* XP_747952 | 74  74  34 | (Schrettl et al., 2004)  (Greenshields et al., 2007)  (Schrettl et al., 2010) |

*Percentage identity between the deduced amino acid sequences of the identified gene and the closest characterised BLAST hit
