# Supplementary material for: An Extracellular Siderophore Is Required to Maintain the Mutualistic Interaction of Epichloë festucae with Lolium perenne
Source: PLoS Pathog. 2013 May 2;9(5):e1003332. doi: 10.1371/journal.ppat.1003332 (PMC3642064; doi:10.1371/journal.ppat.1003332)
Supplement: Table S2 — Iron-Responsive Genes in E. festucae. (DOCX) [file ppat.1003332.s005.docx]

**Supplemental Table 2.** Iron-Responsive Genes in *E. festucae*

| Gene Name | Putative function | Closest characterised  BLAST hit | Percentage  identity* | References |
| --- | --- | --- | --- | --- |
| *ftrA*  *fetC*  *hapX* | high-affinity iron permease  ferrioxidase  bZIP-type regulator | *Aspergillus fumigatus* AAT84596  *Gibberella zeae* XP_385335  *Aspergillus fumigatus* XP_747952 | 74  74  34 | (Schrettl et al., 2004)  (Greenshields et al., 2007)  (Schrettl et al., 2010) |

*Percentage identity between the deduced amino acid sequences of the identified gene and the closest characterised BLAST hit
